# Profiling plasma protease activity with charge-changing peptides enables detection and classification of gastrointestinal cancers

**DOI:** 10.1038/s41598-025-17915-0

**Published:** 2025-09-01

**Authors:** Thanawat Suwatthanarak, Florian Goncalves, Pariyada Tanjak, Kullanist Thanormjit, Amphun Chaiboonchoe, Onchira Acharayothin, Phattarapon Sonthi, Tharathorn Suwatthanarak, Thammawat Parakonthun, Jirawat Swangsri, Asada Methasate, Prasert Auewarakul, Melissa H. Wong, Jared M. Fischer, Vitoon Chinswangwatanakul

**Affiliations:** 1 Siriraj Cancer Center, Faculty of Medicine Siriraj Hospital, Mahidol University, Bangkok, 10700 Thailand; 2 Department of Surgery, Faculty of Medicine Siriraj Hospital, Mahidol University, Bangkok, 10700 Thailand; 3 Knight Cancer Institute, Oregon Health & Science University, Portland, OR 97239 USA; 4 Cancer Early Detection Advanced Research Center, Oregon Health & Science University, Portland, OR 97201 USA; 5 Department of Pharmacology, Faculty of Medicine Siriraj Hospital, Mahidol University, Bangkok, 10700 Thailand; 6 Department of Bioinformatics and Genomics, Graduate School of Advanced Preventive Medical Sciences, Kanazawa University, 13-1, Takaramachi, Kanazawa, Ishikawa 920-8641 Japan; 7 Department of Microbiology, Faculty of Medicine Siriraj Hospital, Mahidol University, Bangkok, 10700 Thailand; 8 Department of Cell, Developmental and Cancer Biology, Oregon Health & Science University, Portland, OR 97239 USA; 9 Department of Molecular and Medical Genetics, Oregon Health & Science University, Portland, OR 97239 USA

**Keywords:** Colorectal adenocarcinoma, Esophagogastric junction adenocarcinoma, Gastric adenocarcinoma, Peptide, Plasma, Protease, Biochemistry, Biological techniques, Cancer, Chemical biology, Gastroenterology, Medical research, Oncology

## Abstract

Early detection of gastrointestinal (GI) cancers—including colorectal cancer (CRC), gastric cancer (GC), and esophagogastric junction cancer (EGJC)—is essential for improving patient outcomes. However, current diagnostic methods such as endoscopy and colonoscopy are invasive, costly, and not widely accessible. Proteases are elevated in many cancers and are detectable in peripheral blood, making them promising candidates for noninvasive diagnostic strategies. We employed a six-probe charge-changing peptide (CCP) panel to profile cancer-associated protease activity in human plasma. Each CCP undergoes a charge shift upon cleavage by a specific protease, enabling detection via gel electrophoresis. Plasma samples from GI cancer patients (CRC, GC, EGJC; *N* = 68) and healthy controls (HC; *N* = 31) were analyzed. Protease activity profiles were analyzed using statistical tests, principal component analysis, and binary logistic regression (LR) models trained on the most informative probes. Model performance was evaluated through repeated cross-validation. Distinct protease activity profiles were observed among CRC, upper GI cancers (UGIC; GC + EGJC), and HC groups. Probe designed to be cleaved by cathepsin B showed the strongest discrimination between cancer and control samples, while probes designed to be cleaved by ubiquitin-specific peptidase 15 and plasmin were identified as the most informative subtype-specific markers for UGIC and CRC, respectively. LR models built on these single probes demonstrated excellent diagnostic performance, with AUCs exceeding 0.95, and both sensitivity and specificity greater than 90%. Our findings highlight CCP-based protease profiling as a minimally invasive, accurate, and scalable method for GI cancer detection and classification. This platform holds strong potential for clinical application in cancer screening, pending further validation in larger, independent cohorts.

**Supplementary Information:**

The online version contains supplementary material available at 10.1038/s41598-025-17915-0.

## Introduction

Gastrointestinal (GI) cancers—including colorectal cancer (CRC), gastric cancer (GC), and esophagogastric junction cancer (EGJC)—remain a major global health challenge, accounting for a significant proportion of cancer-related morbidity and mortality^1,2^. CRC is the third most common cancer worldwide, while GC remains the fifth most common and fourth leading cause of cancer deaths^3,4^. The prognosis of these cancers is strongly dependent on early detection, as five-year survival rates drop substantially with advanced-stage diagnosis^3–5^. Unfortunately, current screening tools such as colonoscopy and upper endoscopy, although effective, are invasive, costly, and often inaccessible, particularly in low-resource settings^6,7^. As a result, there is a need for accurate, noninvasive, and accessible diagnostic methods to improve early detection and patient outcomes.

Emerging liquid biopsy technologies have opened new avenues for cancer diagnostics by enabling the detection of tumor-derived biomarkers in bodily fluids such as blood^8^. While most efforts have focused on circulating nucleic acids or exosomes^9,10^, circulating proteases represent an underexplored but highly promising class of biomarkers^11^. Proteases are actively involved in cancer progression through their roles in extracellular matrix remodeling, invasion, metastasis, angiogenesis, and immune evasion^12–14^. Critically, changes in protease activity—rather than abundance alone—may better reflect dynamic biological processes associated with malignancy, making them attractive candidates for functional biomarker development^15^.

The charge-changing peptide (CCP) assay has shown promise as a method for measuring circulating protease activity^16^. To capture protease activity directly from plasma, we employed a panel of six CCP probes that were designed to both be cleaved by a diverse array of proteases and also target specific cancer-relevant proteases, including angiotensin-converting enzyme 2 (ACE2), cathepsin B (CATB), methionine aminopeptidases 1 and 2 (METAP1/2), matrix metalloproteinase 14 (MMP14), plasmin, and ubiquitin-specific peptidase 15 (USP15)^16–22^. The six selected proteases—ACE2, CATB, METAP1/2, MMP14, plasmin, and USP15—were prioritized based on their established roles in GI cancer biology and their representation of diverse enzymatic classes, including metalloproteases, cysteine proteases, serine proteases, and deubiquitinases. These enzymes have been implicated in key cancer-associated processes such as extracellular matrix degradation (MMP14, plasmin), tumor invasion and metastasis (CATB), immune regulation and signaling (USP15), and cancer-related proteolytic processing (ACE2, METAP1/2). Importantly, their dysregulated activity has been reported in CRC, GC, and EGJC, making them compelling candidates for inclusion in a focused, functional protease activity panel for cancer detection^16–22^. Each CCP probe is engineered to undergo a net charge shift upon enzymatic cleavage, enabling detection via simple gel electrophoresis^16^. This method requires minimal sample volume, enables rapid analysis, and offers a cost-effective, scalable, and minimally invasive platform for assessing protease activity in clinical specimens.

Herein, we applied the CCP panel to plasma samples from patients with CRC, GC, or EGJC, along with healthy controls (HC), to identify disease-specific proteolytic signatures. We analyzed the activity profiles using statistical methods, dimensionality reduction, and logistic regression (LR) modeling to classify cancer subtypes and evaluate diagnostic performance. Key proteases—plasmin, USP15, and CATB—emerged as robust predictors for CRC, upper GI cancers (UGIC; GC + EGJC), and pooled GI cancers, respectively. Altogether, our study shows a functional, protease-based assay with strong potential to support non-invasive detection and classification of GI cancers.

## Materials and methods

### Sample collection and preparation

At Siriraj Hospital, blood samples were collected from patients prior to receiving any treatment. Five milliliters of blood were drawn into heparin tubes (BD, Franklin Lakes, NJ, USA) from patients diagnosed with esophagogastric junction, gastric, or colorectal adenocarcinoma (*N* = 68), as well as from HCs (*N* = 31) who underwent endoscopic check-ups or were healthy volunteers with no abnormalities in the GI tract. Following collection, the blood samples were centrifuged at 1500 × g for 15 min to separate the plasma. The plasma samples were stored at − 80 °C until further use. Total protein concentrations in the plasma were quantified via the Bradford assay (Bio-Rad Laboratories Inc, Hercules, CA, USA) according to the manufacturer’s instructions. With respect to ethical considerations, the study complied with guidelines governing human research. Informed consent was obtained from all participants, and the procedures were approved by the Siriraj Institutional Review Board (COA no. Si 698/2022). The authors confirm that all experiments were performed in accordance with the relevant guidelines and regulations.

For plasmin solution, lyophilized native human plasmin protein (active) (ab90928; Abcam Ltd, Cambridge, UK) was reconstituted in ultrapure water (Milli-Q Advantage A10; Merck Millipore, Burlington, MA, USA). The reconstituted protein was then diluted in phosphate-buffered saline to the desired concentration.

### CCP preparation

Peptide sequences and cleavage sites for the CCPs were designed using the MEROPS and ProCleave database platforms, as previously reported (Table 1)^16,23,24^. Representative mass spectrometry (MS)-based validation of CCP cleavage products for these probes was performed in our previous study on pancreatic cancer^16^, demonstrating that enzymatic digestion occurred near the intended substrate sites. CCP probes were synthesized by Bio Basic Canada Inc (Markham, ON, Canada; Supplementary File 1). To conjugate the fluorescent dye to the peptides, a 100 mM sodium bicarbonate solution was prepared by dissolving 0.8401 g of sodium bicarbonate in 100 mL of ultrapure water and adjusting the pH to 8.2. A peptide solution was then made by dissolving 5 mg of peptide in 500 µL of the sodium bicarbonate buffer, yielding a 10 mg/mL concentration. Concurrently, a 10 mg/mL solution of BODIPY FL NHS ester (Lumiprobe Ltd, Wan Chai, Hong Kong) was prepared by dissolving 5 mg of the dye in 500 µL of dimethyl sulfoxide. Equal volumes of the peptide and dye solutions (500 µL each) were mixed and incubated for 1 h at room temperature in the dark to facilitate conjugation. After incubation, 150 µL of the conjugated solution was aliquoted into 10 tubes (15 µL per tube) and stored at − 20 °C for future use. For working stock preparation, 150 µL of the conjugated solution was diluted 1:10 by mixing with 1.35 mL of ultrapure water, resulting in a 500 µg/mL solution. This diluted solution was aliquoted into 30 tubes (50 µL each) for subsequent experimental use.

**Table 1:** CCP probes, target proteases, peptide sequences, and charge variations.

Probe (target)	Peptide sequence (/=cleavage site)	Charge
Probe 1 (ACE2)	Ac-N-GEPEP/FAGAGK-(Bodipy)-CO-NH2	− 2 to + 1
Probe 2 (CATB)	Ac-N-DGLA/GGAGGK-(Bodipy)-CO-NH2	− 1 to + 1
Probe 3 (METAP1/2)	Ac-N-DGDGMAR/TLK-(Bodipy)-CO-NH2	− 1 to + 2
Probe 4 (MMP14)	Ac-N-DGDPAG/LRGAGK-(Bodipy)-CO-NH2	− 1 to + 2
Probe 5 (plasmin)	Ac-N-DGDPSLRS/VSGK-(Bodipy)-CO-NH2	− 1 to + 1
Probe 6 (USP15)	Ac-N-DGDLRG/GMPGSGK-(Bodipy)-CO-NH2	− 1 to + 1

### Protease activity assay

Protease assays were conducted in a 96-well V-bottom plate. Before starting, the prepared CCPs were centrifuged and vortexed to ensure proper mixing. Each well received 2 µL of 50 mM calcium chloride, which was followed by 4 µL of the working stock peptide solution. Subsequently, 4 µL of plasma (containing 16 µg of protein per µL) was added to each well. The plate was sealed and incubated on a shaker at room temperature (300 rpm) for 45 min in the dark to allow the reactions to proceed. Following incubation, 8 µL of the reaction mixture was loaded onto a 20% acrylamide TBE gel (Novex TBE Gels, 20%; Thermo Fisher Scientific Inc, Waltham, MA, USA). Electrophoresis was performed at 300 V for 60 min, with the polarity inverted to ensure migration toward the negative electrode. After electrophoresis, the gel was imaged via a biomolecular imager (ImageQuant LAS 4010; GE Healthcare, Chicago, IL, USA) for fluorescence detection using a SYBR Green filter with a 240 ms exposure time. To assess protease activity, band intensities were quantified with ImageJ software (National Institutes of Health, Bethesda, MD, USA).

### Data preprocessing and normalization

Protease activity data (band intensities) were transformed into z-scores to enable standardized comparisons across different groups. Z-score transformation was calculated for each probe’s dataset by subtracting the mean value of the dataset from individual raw values and dividing by the dataset’s standard deviation.

### Group comparison analysis

Non-parametric Kruskal-Wallis tests were performed to compare protease activity distributions across four clinical groups (HC, CRC, EGJC and GC). We selected this non-parametric test due to non-normal distribution of the data and the presence of multiple independent groups. Following significant Kruskal-Wallis results (*p* < 0.05), we performed pairwise comparisons using Dunn’s test with Bonferroni adjustment to control for family-wise error rate while determining specific group differences. Statistical significance was established at *p* < 0.05, with significance levels indicated as: *p < 0.05, **p < 0.01, ***p < 0.001, ****p < 0.0001.

### Dimensionality reduction

Principal component analysis (PCA) was conducted to identify patterns within the multivariate protease dataset. PCA was performed using the prcomp() function in R, with parameters set to center and scale the data. To visualize clustering patterns among clinical groups, 95% confidence ellipses were overlaid in PCA space to indicate the uncertainty in the estimated group centroids. These ellipses were computed under the assumption of multivariate normality using each group’s mean and covariance matrix, based on the six z-score–normalized protease activity markers. Importantly, the ellipses do not enclose 95% of the individual data points but represent confidence regions for the true group centroids. Ellipses were generated using the fviz_pca_ind() function from the factoextra R package, with parameters addEllipses = TRUE, ellipse.type = “confidence”, and ellipse.level = 0.95. This approach is statistically equivalent to ellipse generation via the stat_ellipse() function from ggplot2, which similarly accounts for group size and covariance.

### Classification model development and logistic regression

Separate binary classification models were developed to distinguish (1) UGIC (EGJC + GC) from HC, and (2) CRC from HC. EGJC and GC were grouped due to their close biological and physiological similarity. For each model, predictor selection was guided by permutation importance analysis based on 100 random permutations of the accuracy metric. LR was used for model training, incorporating k-fold cross-validation with random repeats to ensure reliability. Class imbalance between cancer and control groups was addressed by applying weighted observations where appropriate.

### Model validation and performance assessment

Model performance was evaluated using nested cross-validation with 3-fold partitioning repeated 200 times for binary LR models. For each fold, the following metrics were calculated and saved: area under the receiver operating characteristic curve (AUC-ROC), sensitivity (true positive rate, TPR), specificity (true negative rate, TNR) and accuracy. We reported these performance metrics as average values across all resampled folds with their respective standard deviation as a variability metric across folds. For visualization, individual ROC curves from each resampling iteration were plotted alongside the mean ROC curve.

### Patient pooling for binary logistic regression model development

To establish different binary models with enough samples to ensure statistical relevance and robustness, we pooled clinical groups in new nested datasets. This grouping strategy allowed us to combine patients into new groups for each step of the analysis. We first combined EGJC and GC groups to form the UGIC group, and developed a model to classify patients in this group versus control. Subsequently, we merged CRC with EGJC and GC to create the GI cancer group, and developed a fully binary model GI cancer cohort versus healthy cohort.

### Visualization methods

Visualization of our statistical analysis results was performed using Boxplots for protease activity across clinical groups with statistical significance visualization, Radar plots to highlight multivariate protease profiles across groups, PCA biplots with 95% confidence ellipses to visualize sample clustering. ROC curves with AUC were used to evaluate the performance of our classification models.

### Statistical power analysis

Statistical power analyses were conducted in R (version 4.4.1) using the pwr package (version 1.3-0). For two-group comparisons, the pwr.t.test() function was employed with Cohen’s d as the effect size metric, where d ≈ 0.5 indicates a medium effect. For comparisons involving four groups, one-way ANOVA was used via the pwr.anova.test() function, applying Cohen’s f, with f ≈ 0.25 denoting a medium effect. Both prospective and post-hoc power calculations were performed, with power ≥ 0.8 considered adequate. Post-hoc estimates were interpreted cautiously, recognizing their reliance on observed effect sizes and limited inferential value for study design. For cancer vs. control comparisons (*N* = 68 vs. *N* = 31), a prospective power analysis using a two-sample t-test indicated that the study had 80% power to detect an effect size of d = 0.72, representing a moderately large effect. This confirms the study’s strong capability to detect the substantial effects observed for several biomarkers (most with Cohen’s d > 1.0), in line with existing biomarker literature. For four-group comparisons (EGJC, CRC, GC, and controls), a power analysis using one-way ANOVA, based on average group sizes of ~ 25, yielded 52% power to detect a medium effect (f = 0.25). Although this falls below the ideal 80% threshold, it still provides meaningful sensitivity given the large observed effect sizes (Cohen’s d: 1.03–2.35). Post-hoc calculations based on these large effects yielded power estimates > 95%. Overall, both prospective and post-hoc analyses support that the study is adequately powered to detect large effects, which were consistently observed. Nonetheless, future studies with larger and more balanced cohorts will be essential for robust detection of smaller intergroup differences, particularly among cancer subtypes.

### Software and packages

All analyses were performed in R (version 4.4.1). Key packages used for statistical analysis included: stats for statistical testing, FactoMineR and factoextra for PCA, pROC for ROC analysis, ggplot2 for data visualization, rstatix for Dunn’s test and caret for model training and validation^25–31^.

### Enzyme-linked immunosorbent assay

To validate the plasmin identified through the CCP assay, we performed an enzyme-linked immunosorbent assay (ELISA) via a Human Plasmin ELISA Kit (catalog number RE3112H; Reed Biotech, Wuhan Optics Valley Biolake, Hubei, China). All reagents, working standards, and samples were equilibrated to room temperature (25 °C) before use. We added 100 µL of plasmin standard or plasma sample, each at a concentration of 1 µg/µL, to each well. The wells were sealed and incubated for 90 min at 37 °C. Following this initial incubation, the wells were manually washed three times with 300 µL of 1× Wash Buffer. Next, 100 µL of 1× biotinylated detection antibody working solution was added to each well, and the plate was incubated for 1 h at 37 °C. Following another wash step, we added 100 µL of 1× horseradish peroxidase conjugate working solution and incubated it for 30 min at 37 °C. After incubation, the wells were washed again, and 100 µL of substrate reagent was added to each well. The plate was then incubated for 15 min at 37 °C until a blue color developed. The reaction was stopped by adding 50 µL of stop solution, changing the color from blue to yellow. The absorbance was measured at 450 nm using a microplate reader (Synergy H1; BioTek, Winooski, VT, USA).

### Mass spectrometry analysis

MS analysis was conducted to validate the results of protease identification. Sample preparation began by precipitating 100 µL of serum with 1000 µL of 1% trichloroacetic acid in isopropanol. The protein concentration was measured via the Bradford assay and adjusted to 10 mg/mL. The proteins were reduced with 10 mM dithiothreitol for 30 min at 65 °C and then alkylated with 25 mM iodoacetamide for 20 min at room temperature. Protein digestion was performed by adding 2 µg of trypsin and incubating for 16 h. The digestion was stopped with the addition of 1% formic acid, and the peptide mixture was centrifuged at 14 000 rpm for 10 min. The supernatant was collected for MS analysis.

The samples were analyzed via an Agilent QTOF 6545XT LC‒MS system (Agilent Technologies Inc, Santa Clara, CA, USA). A 10 µL injection was made onto an Agilent Peptide Map column (2.1 × 150 mm, 2.7 μm) at 60 °C. The chromatographic run time was 85 min, with a flow rate of 0.4 mL/min. Mass spectra were acquired in the mass range of 100–1700 m/z in MS/MS centroid mode with positive ionization. The MS conditions were as follows: gas temperature, 325 °C; drying gas flow, 13 L/h; nebulizer pressure, 35 psi; capillary voltage, 4000 V; and nozzle voltage, 500 V. The MS scan range was set from 40 to 1700 m/z, and the MS/MS scan range was from 25 to 1000 m/z. The collision energy varied between 10 and 30 eV depending on the peptide charge. The reference mass was set to 922.0098 m/z.

Data processing was performed using MSconvert and OpenMS. Peptide identification was performed with MaxQuant software version 2.6.3^32^. The search was conducted against the UniProt protein database, focusing on plasmin.

## Results

### CCP assay

**Fig. 1:**
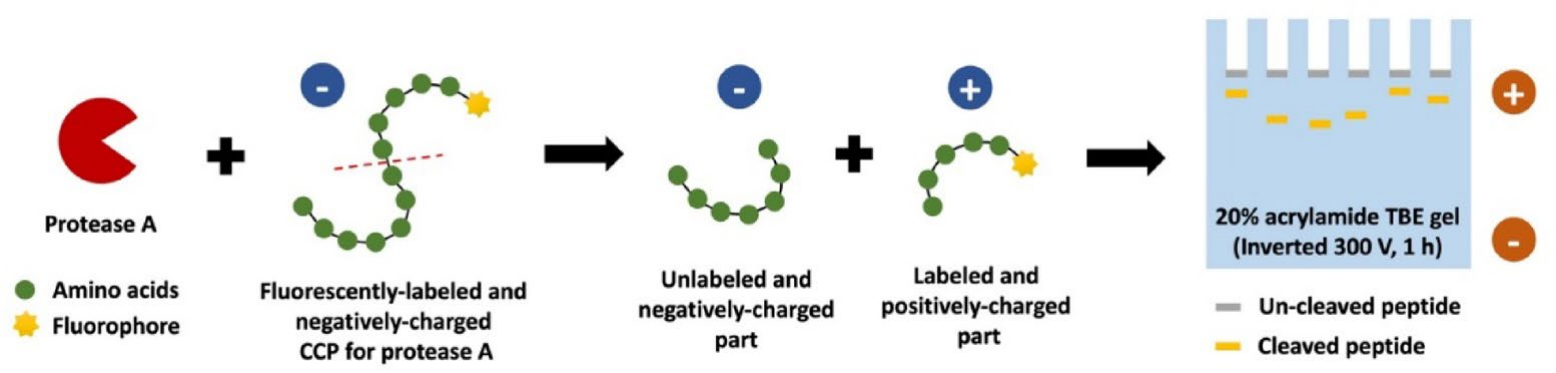
Schematic representation of the charge-changing peptide assay. The charge-changing peptide (CCP) assay was designed to undergo charge variation upon interaction with a target protease, resulting in cleavage that produces one negatively charged, and one positively charged fragment. The positively charged fragment, labeled with a fluorescent marker, serves as the primary detection indicator. Plasma samples were incubated with the CCP for 45 min before gel electrophoresis, which separated and facilitated detection of the labeled peptides.

This study aimed to identify cancer-associated proteases in plasma samples from patients diagnosed with colorectal, gastric, or esophagogastric junction adenocarcinoma. We employed a panel of six fluorescently labeled CCP probes that were designed for cleavage by specific proteases implicated in cancer progression: ACE2, CATB, METAP1/2, MMP14, plasmin, and USP15^16–22^. Peptide sequences and cleavage sites for the CCP design were identified using the MEROPS and ProCleave databases^23,24^. Each probe was engineered to undergo charge variation upon interaction with its target protease, shifting from a negative charge to one negatively charged fragment and one positively charged fragment (Fig. 1). However, due to the promiscuous nature of proteases, off-target cleavage may also occur. The labeled, positively charged fragment served as the key detection marker in the assay (Fig. 1). The experimental procedure involved mixing 4 µL of plasma with 4 µL of CCP and incubating for 45 min (Fig. 1). This was followed by gel electrophoresis to separate the cleaved and labeled peptides (Fig. 1). The fluorescence signal of the cleaved peptide, corresponding to protease activity, was imaged and analyzed with ImageJ software. This approach aimed to reveal distinct protease signatures associated with cancer.

To assess assay specificity and sensitivity, we selected plasmin—a well-characterized, commercially available serine protease—as a representative target. Using the prepared protease standards, we demonstrated that the CCP probe for plasmin exhibited high selectivity with minimal cross-reactivity toward other proteases in the panel, confirming its specificity for detecting plasmin activity (Fig. S1A and B). Both the MMP14 and METAP1/2 probes showed minimal reactivity, as expected, due to the presence of arginine residues in suboptimal cleavage positions (Fig. S1A and B). We also observed a positive correlation between signal intensity and plasmin concentration in the prepared samples (Fig. S1C and D), indicating the probe’s sensitivity to varying levels of plasmin. Notably, the lowest detectable concentration was < 1 nM, showing the assay’s high sensitivity. These findings support the CCP probe as a selective and sensitive tool for detecting plasmin activity in biological samples.

### Protease activity profiling in human plasma samples using a 6-probe CCP panel

**Fig. 2:**
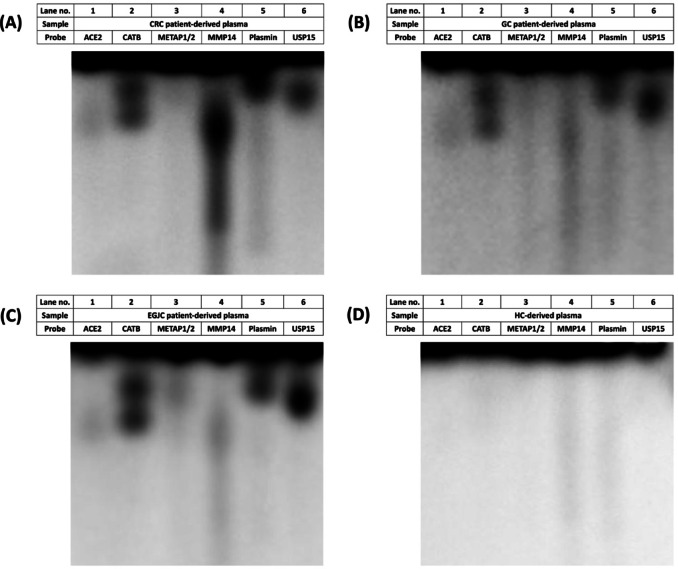
Fluorescence images of protease activity profiles across clinical groups using a 6-probe CCP panel. Representative gel fluorescence images display protease activity profiles in plasma samples from patients with (**A**) colorectal cancer (CRC), (**B**) gastric cancer (GC), (**C**) esophagogastric junction cancer (EGJC), and (**D**) healthy controls (HC). Marked differences in protease activity are evident between cancer patients and healthy individuals.

We next evaluated protease activity in human plasma samples using our panel of six CCP probes across four clinical groups: HC, CRC, EGJC, and GC. Participant demographics are summarized in Table S1. Gel fluorescence imaging revealed distinct proteolytic activity patterns corresponding to each probe, enabling the visualization of cancer-associated protease activity in plasma samples (Fig. 2, Supplementary File 2). Notably, clear differences in protease profiles were observed between cancer patients and healthy individuals, with cancer samples exhibiting significantly elevated activity signatures (Fig. 2). These initial observations suggest that the CCP panel can capture disease-associated protease dysregulation in a clinically relevant manner.

**Fig. 3:**
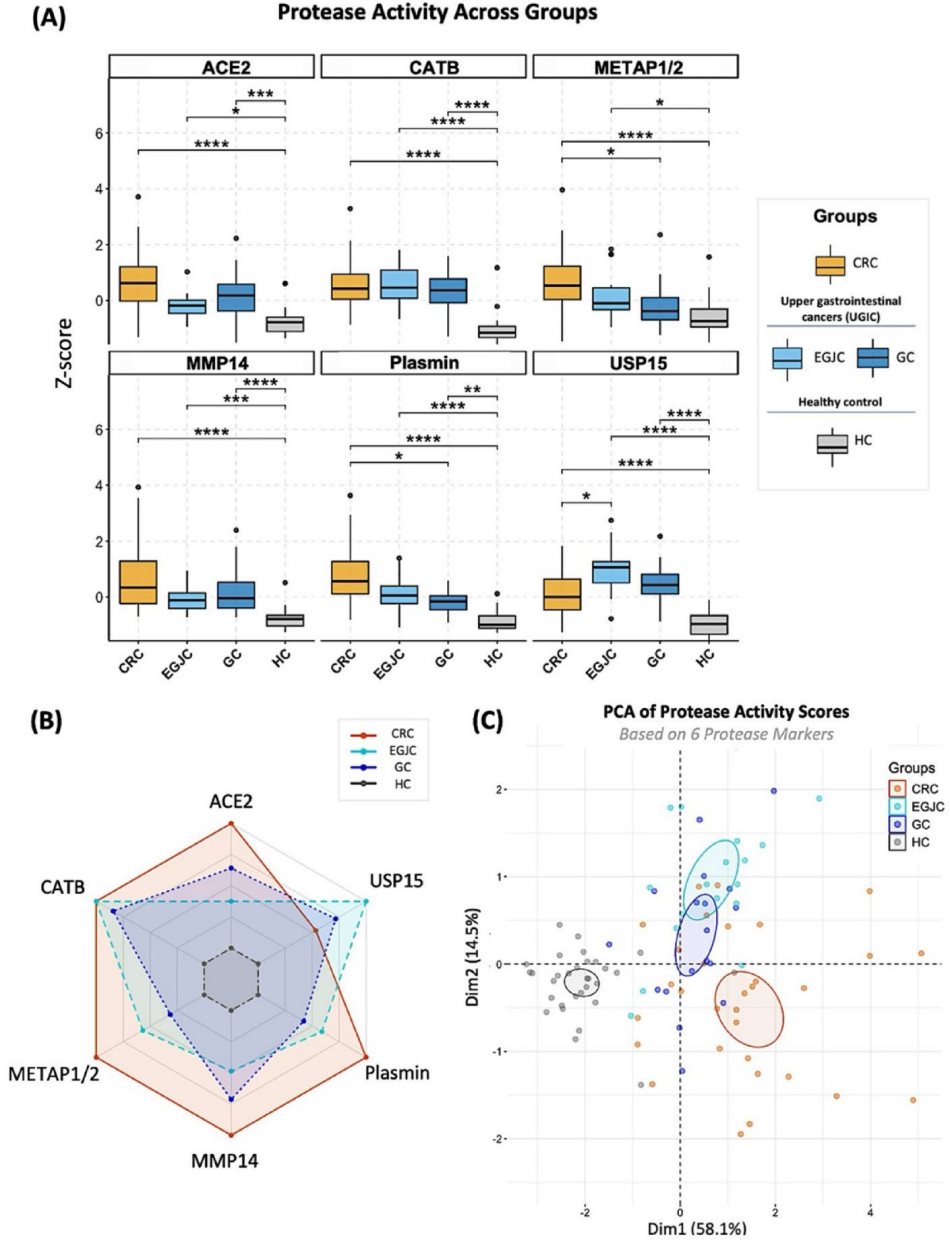
Protease activity profiles across clinical groups using a 6-probe CCP panel. (**A**) Boxplots of protease activity z-scores measured with 6 different probes and across 4 different clinical groups: healthy control (HC), colorectal cancer (CRC), esophagogastric junction cancer (EGJC), and gastric cancer (GC). Statistical comparisons were performed using Kruskal–Wallis tests with Bonferroni correction for multiple comparisons. Post hoc pairwise differences were assessed using Dunn’s test with Bonferroni adjustment (*p < 0.05, **p < 0.01, ***p < 0.001, ****p < 0.0001). Sample sizes: HC (*N* = 31), CRC (*N* = 32), EGJC (*N* = 18), and GC (*N* = 18). (**B**) Radar plot showing z-score distribution of protease activity as measured with 6 different probes, across four clinical groups (HC, CRC, EGJC, GC). (**C**) Principal component analysis (PCA) projection of protease activity profiles (6 probes) in a reduced 2-dimensional space, illustrating clustering patterns among 4 clinical groups: HC (grey), CRC (orange), EGJC (turquoise), and GC (navy blue). Points represent individual plasma samples, with 95% confidence ellipses indicating group clustering. The first two principal components explain 58.1% (PC1) and 14.5% (PC2) of the total variance. Data were standardized (mean = 0, SD = 1) prior to PCA.

As a result of statistical analysis, significant differences in probe signals and distinct protease activity signatures were observed among the four clinical groups (Fig. 3A). All probes, except METAP1/2, showed significant signal differences between each cancer group (CRC, EGJC or GC) and the HC group (p-values ranging from < 0.0001 and < 0.05) (Fig. 3A). The CRC group consistently demonstrated the strongest statistical difference compared to the control group (*p* < 0.0001) across all 6 probes (Fig. 3A). No significant differences were observed between EGJC and GC groups for any of the tested probes (Fig. 3A). These two groups showed similarly strong statistical differences compared to the HC group for most predictors (CATB, MMP14, Plasmin and USP15; *p* < 0.01), supporting their combination into a single “UGIC” group for further analysis (Fig. 3A).

Radar plot visualization summarized unique protease activity signatures across groups (Fig. 3B). CRC showed broad up-regulation of five probes, with lower USP15 signal compared to EGJC and GC groups (Fig. 3B). The HC group demonstrated the lowest protease activity across all measured probes (Fig. 3B). PCA revealed partially distinct clustering of clinical groups, with healthy controls separating from cancer groups primarily along PC1, which accounted for 58.1% of total variance (Fig. 3C). To aid visualization, 95% confidence ellipses were plotted around each group centroid, representing uncertainty in the estimated mean position based on group-specific covariance in PCA space. These ellipses, calculated under multivariate normality assumptions from z-score–normalized protease activity markers, do not enclose 95% of individual points. Variability in ellipse shape—especially in the HC group—may reflect mild non-normality and outliers, which were considered in interpreting clustering patterns.

**Fig. 4:**
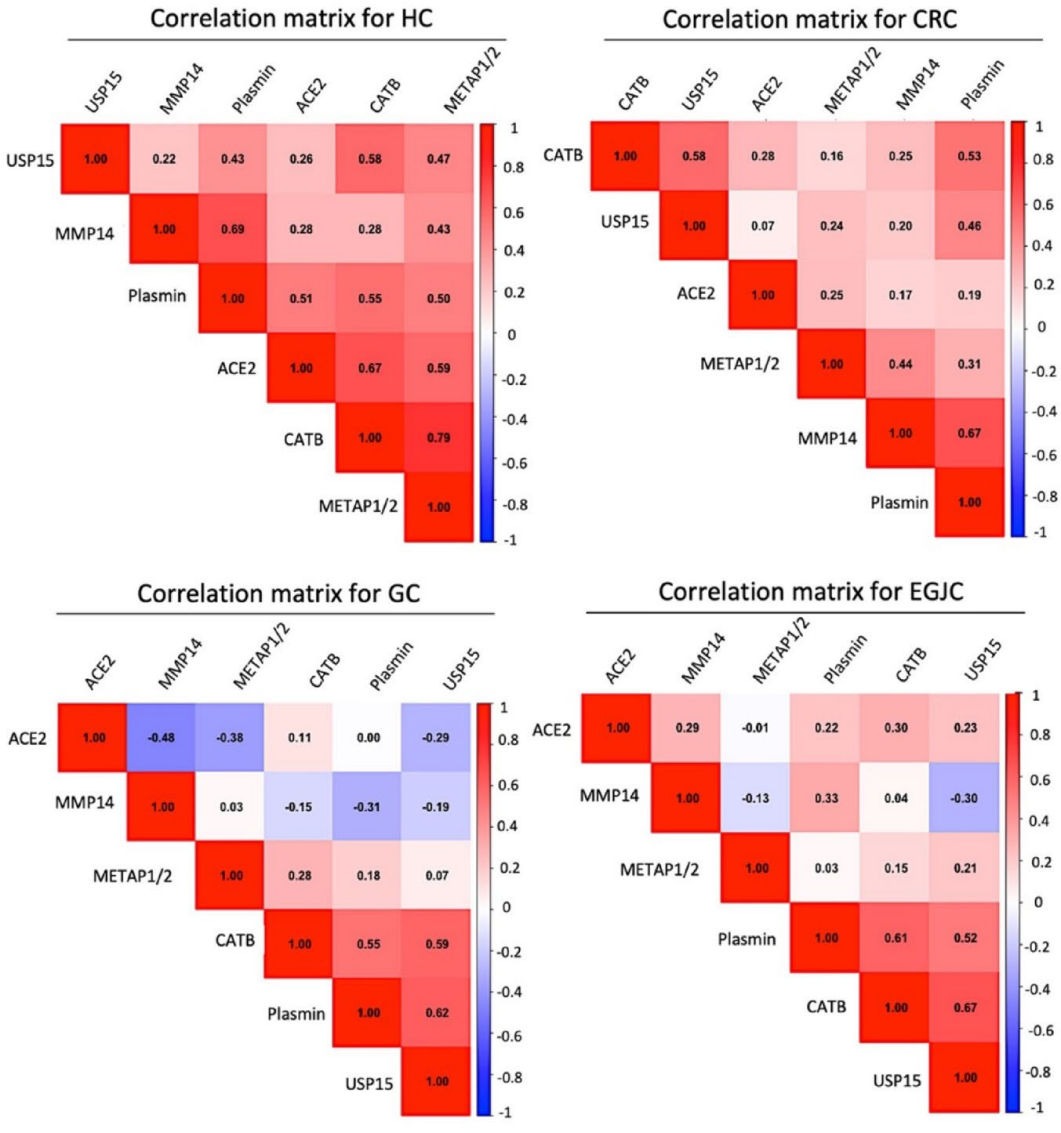
Pairwise Pearson correlations between 6 protease probe predictors. Pearson correlation coefficients were calculated between the six protease probe signals (ACE2, CATB, METAP1/2, MMP14, plasmin, and USP15) for each group: healthy control (HC), colorectal cancer (CRC), gastric cancer (GC), and esophagogastric junction cancer (EGJC). Heatmaps were generated via the cor() function in R, providing a measure of linear association between variables. Color gradient for correlation pattern intensities: Blue (-1) -> White (0) -> Red (+ 1), representing negative, no, and positive correlations, respectively. For readability purpose, only upper triangle is displayed for each group with annotated correlation coefficient for each pairwise correlation.

To investigate protease co-activity relationships, we calculated pairwise Pearson correlation coefficients between the six CCP probe readouts within each clinical group. This analysis revealed distinct, group-specific protease interaction patterns, visualized as hierarchical clustering heatmaps (Fig. 4). In the HC group, a high degree of coordinated protease activity was observed, with strong correlations between CATB and METAP1/2 (*r* = 0.79) and ACE2 and CATB (*r* = 0.67), suggesting preserved homeostatic regulation (Fig. 4). In contrast, cancer groups displayed more fragmented and cancer-specific co-activity patterns. In the CRC group, strong positive correlations were identified between MMP14 and plasmin (*r* = 0.67) and CATB and USP15 (*r* = 0.58), reflecting a shift in protease interplay under malignant conditions (Fig. 4). Among UGIC—comprising GC and EGJC—a shared USP15–plasmin co-activation pattern was observed (Fig. 4). Additionally, GC demonstrated a pronounced anti-correlation between ACE2 and MMP14 (*r*=–0.48), while EGJC exhibited a strong USP15–CATB correlation (*r* = 0.67) and moderate anti-correlation between USP15 and MMP14 (*r*=–0.30) (Fig. 4). Across all cancer groups, METAP1/2 consistently showed weak or negative correlations with other proteases (Fig. 4). Despite lower absolute protease activity levels, the HC group exhibited higher global correlation connectivity (mean *r* = 0.52) compared to cancer groups (mean *r* = 0.38), supporting the notion that cancer disrupts coordinated protease regulation (Fig. 4). This analysis highlights cancer-specific protease co-activation patterns and underscores the potential of multi-protease correlation profiling to identify interaction signatures that extend beyond individual protease activity levels.

Moreover, the identification of plasmin through the CCP assay prompted further validation via ELISA and MS techniques. Consistent with the results of the CCP assay, the ELISA results confirmed elevated plasmin levels in plasma samples from cancer patients compared with those from healthy controls (Fig. S2). In the MS analysis, elevated plasmin level in cancer patient plasma was also observed (Fig. S2). These validation studies further confirm the reliability of plasmin as a potential biomarker, reinforcing the utility of CCP-based protease profiling for cancer diagnostics. The agreement between these methods emphasizes the robustness of the CCP assay in identifying clinically relevant protease activities.

Collectively, these results reveal intriguing insights into the protease activity profiles across various cancer types. Notably, the unique signatures of the cancer-associated proteases exhibited distinct variations among the cancer types analyzed. This finding suggests that the CCP assay has the potential to identify the presence of cancer and differentiate between specific cancer types on the basis of their protease activity profiles. Such differentiation could be crucial for tailoring personalized therapeutic strategies and enhancing diagnostic accuracy in clinical settings.

### Logistic regression models for binary classification of upper and lower gastrointestinal cancers

**Fig. 5:**
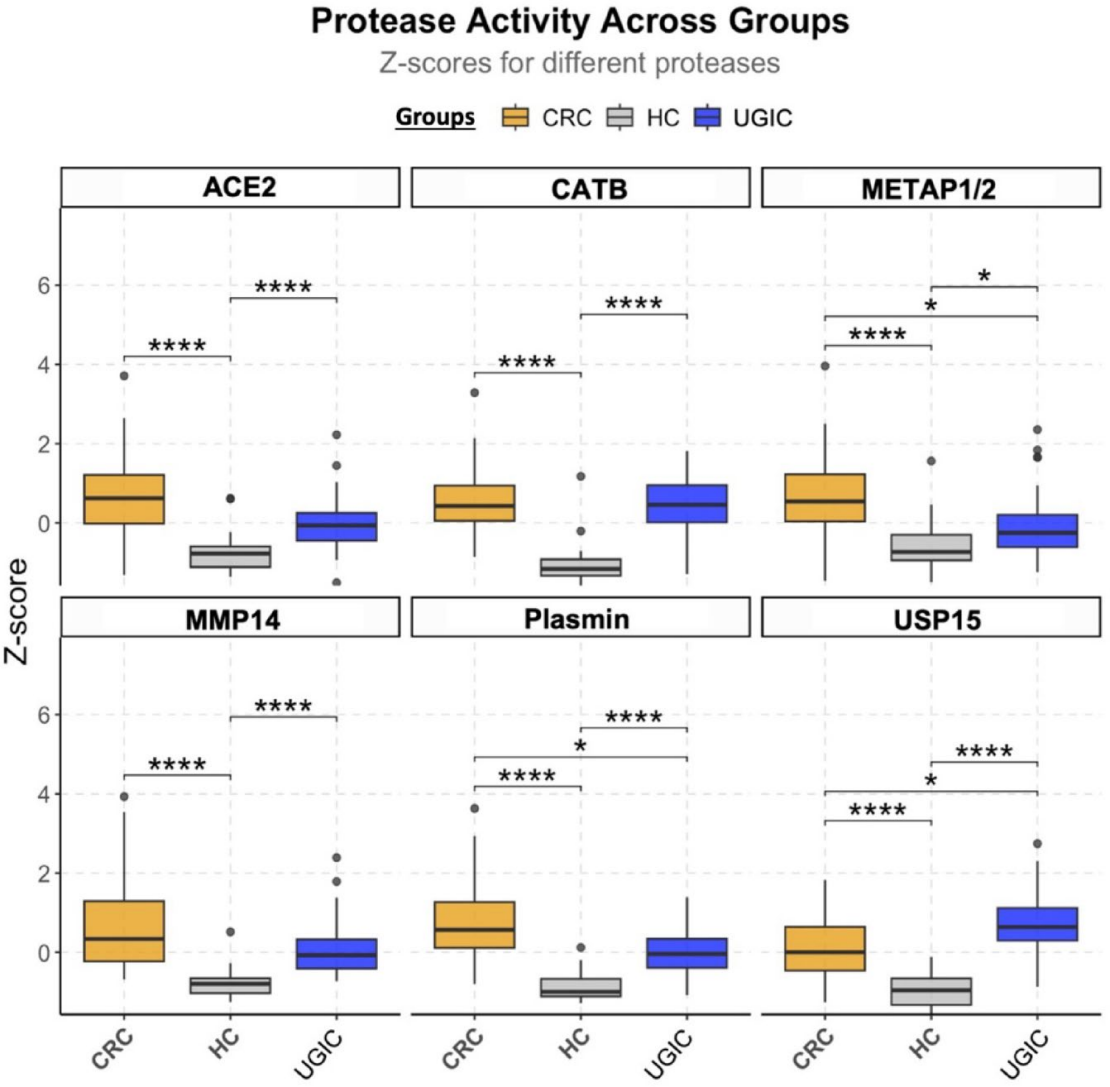
Protease activity profiles across colorectal cancer, upper gastrointestinal cancer, and healthy groups. Boxplots display z-score–normalized protease activity levels measured using six CCP probes across three clinical groups: healthy control (HC; *N* = 31), colorectal cancer (CRC; *N* = 32), and upper gastrointestinal cancer (UGIC; *N* = 36), which include gastric cancer (GC) and esophagogastric junction cancer (EGJC) samples. Statistical comparisons were conducted using the Kruskal–Wallis test with Bonferroni correction to assess overall group differences (*p* < 0.05). Post hoc pairwise comparisons were performed using Dunn’s test with Bonferroni adjustment, with significance thresholds indicated as follows: *p < 0.05, ****p < 0.0001.

Based on the protease activity profiles, plasmin and USP15 probes demonstrated the strongest discriminative potential when comparing the UGIC group with CRC and HC groups (*p* < 0.05 for CRC versus UGIC; *p* < 0.0001 for HC versus CRC/UGIC; Fig. 5). Specifically, plasmin reported activity was higher in the CRC group compared to the UGIC group (*p* < 0.05) and HC (*p* < 0.0001). Conversely, USP15 probe activity was superior in the UGIC group compared to CRC (*p* < 0.05) and HC (*p* < 0.0001) groups (Fig. 5). These support the utility of plasmin and USP15 as subtype-specific protease biomarkers for GI cancer classification.

**Fig. 6:**
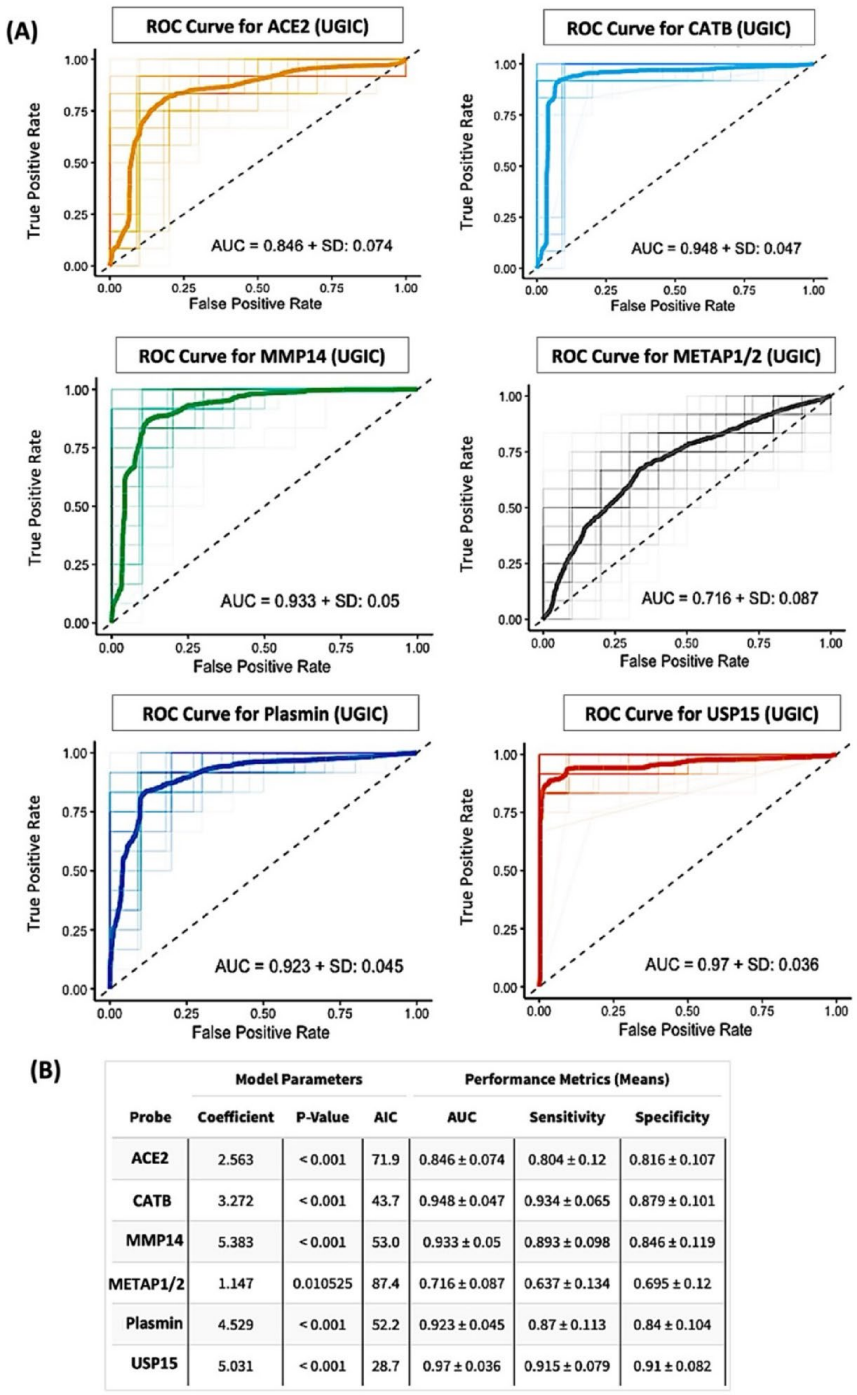
Comparison of single-predictor binary models for classifying upper gastrointestinal cancers. (**A**) ROC curves for logistic regression models using each of the six CCP-derived protease probes individually to classify upper gastrointestinal cancers (UGIC; combining esophagogastric junction cancer and gastric cancer) versus healthy control. Curves represent mean performance across 3-fold cross-validation with 100 random repeats. The USP15 probe yielded the highest classification accuracy, with an AUC of 0.970 ± 0.036. (**B**) Summary table of pooled performance metrics for each single-probe model, including logistic regression coefficients, p-values, Akaike information criterion (AIC), AUC, sensitivity, and specificity. The USP15-based model demonstrated superior diagnostic performance, achieving a sensitivity of 0.915, specificity of 0.910, and the highest AUC among all tested probes.

**Fig. 7:**
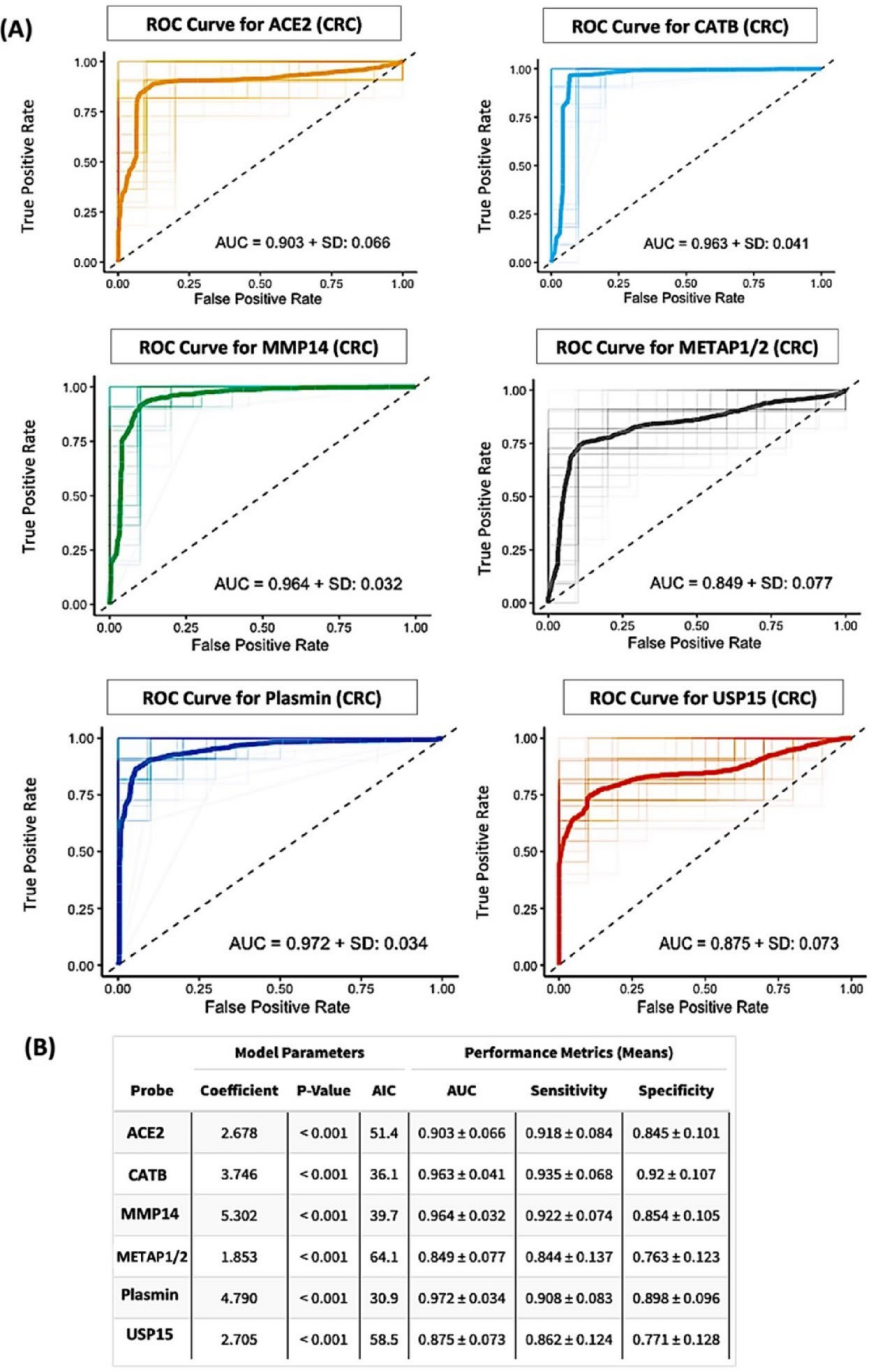
Comparison of single-predictor logistic regression models for classifying colorectal cancer. (**A**) ROC curves for logistic regression models using each of the six CCP-derived protease probes individually to classify colorectal cancer (CRC) versus healthy control. Performance metrics represent the mean of 3-fold cross-validation repeated 100 times. The plasmin probe demonstrated the highest classification performance, achieving an AUC of 0.972 ± 0.034. (**B**) Summary table presenting pooled performance metrics for each single-probe model, including logistic regression coefficients, p-values, Akaike information criterion (AIC), AUC, sensitivity, and specificity. The plasmin-based model yielded the best diagnostic accuracy, with a sensitivity of 0.908, specificity of 0.898, and the highest AUC among all probes tested.

To assess the diagnostic performance of individual protease activity markers, we performed LR modeling followed by ROC curve analysis for each of the six CCP-derived probes across two binary classification tasks: UGIC versus HC and CRC versus HC (Fig. 6 and 7). In the UGIC model (Fig. 6A), USP15 achieved the highest area under the curve (AUC = 0.970 ± 0.036), followed by CATB (0.948 ± 0.047), MMP14 (0.933 ± 0.050), and plasmin (0.923 ± 0.045). In contrast, ACE2 (0.846 ± 0.074) and METAP1/2 (0.716 ± 0.087) showed comparatively lower discriminative ability. Sensitivity and specificity values for the top-performing probes consistently exceeded 0.80, reinforcing their diagnostic utility in UGIC classification (Fig. 6B). For the CRC classification task (Fig. 7A), plasmin demonstrated the highest AUC (0.972 ± 0.034), closely followed by MMP14 (0.964 ± 0.032), CATB (0.963 ± 0.041), and ACE2 (0.903 ± 0.066). Although METAP1/2 and USP15 exhibited slightly lower AUC values (0.849 and 0.875, respectively), all six probes were statistically significant in the logistic models (*p* < 0.001; Fig. 7B). These results indicate that USP15 is the most robust individual classifier for UGIC, while plasmin emerged as the top-performing marker for CRC. Collectively, these findings underscore the potential of CCP-based protease activity profiling as a highly accurate and noninvasive strategy for distinguishing between GI cancer sub-groups.

**Fig. 8:**
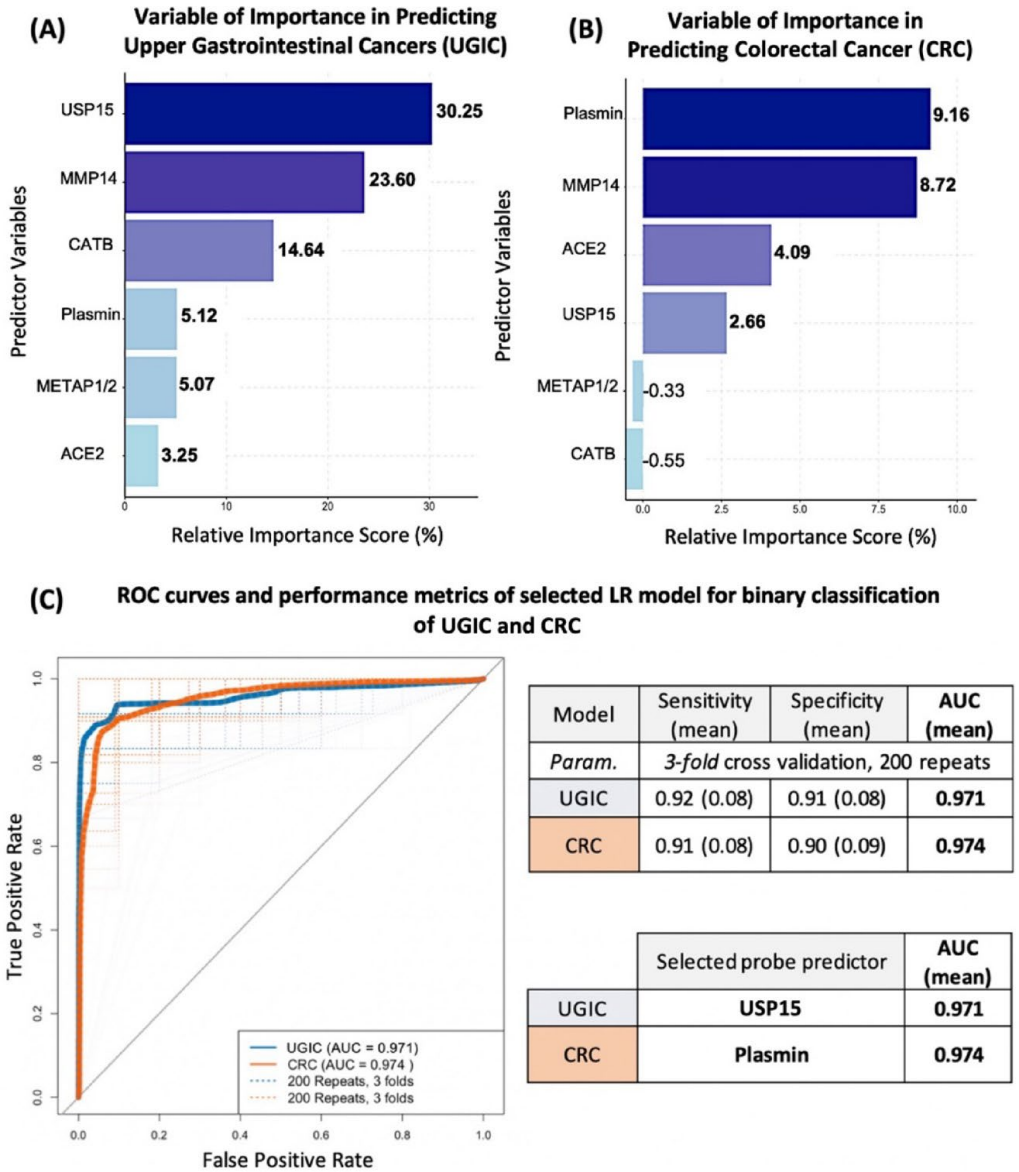
Development and validation of logistic regression models for binary classification of upper gastrointestinal cancers and colorectal cancer. Logistic regression models were developed for binary classification of (i) upper gastrointestinal cancers (UGIC; combining esophagogastric junction cancer and gastric cancer; *N* = 36) versus healthy control (HC; *N* = 31), and (ii) colorectal cancer (CRC; *N* = 32) versus HC (*N* = 31). (**A**,**B**) Permutation Feature Importance Analysis. Predictors were ranked based on mean decrease in model accuracy following 100 random permutations. (**A**) In the UGIC model, USP15 showed the highest relative importance (30.3%), followed by MMP14 and CATB. (**B**) In the CRC model, plasmin was the most influential predictor (9.2%), followed by MMP14 and ACE2. (**C**) Model Performance Evaluation. ROC curves and performance metrics are shown for the selected models using the most predictive individual probe (USP15 for UGIC; plasmin for CRC). Each ROC curve reflects mean AUC values from 3-fold cross-validation repeated 200 times. Both models demonstrated high diagnostic performance, with AUCs of 0.971 (UGIC) and 0.974 (CRC), supporting the clinical utility of these CCP-based biomarkers for cancer classification.

Building upon the performance of individual probes in classifying UGIC and CRC (Fig. 6 and 7), we further examined the contribution of each predictor to the overall logistic regression models using permutation-based importance analysis (Fig. 8). For UGIC, USP15 emerged as the most influential variable, contributing over 30% to the model’s relative importance, followed by MMP14 and CATB (Fig. 8A). In contrast, the CRC model was most strongly driven by plasmin, with MMP14 and ACE2 also contributing substantially (Fig. 8B). These results align with prior single-predictor model analyses, in which USP15 and plasmin achieved the highest individual classification performance for UGIC and CRC, respectively. The final multivariable LR models demonstrated robust diagnostic accuracy, with AUCs of 0.971 for UGIC and 0.974 for CRC, supported by high sensitivity and specificity values across repeated cross-validation (Fig. 8C). Notably, single-marker models using USP15 or plasmin alone yielded AUCs nearly identical to their respective multivariate models, reinforcing the clinical utility of these probes as potential standalone biomarkers in disease-specific protease profiling.

### Logistic regression model for binary classification of gastrointestinal cancer patients and healthy controls

**Fig. 9:**
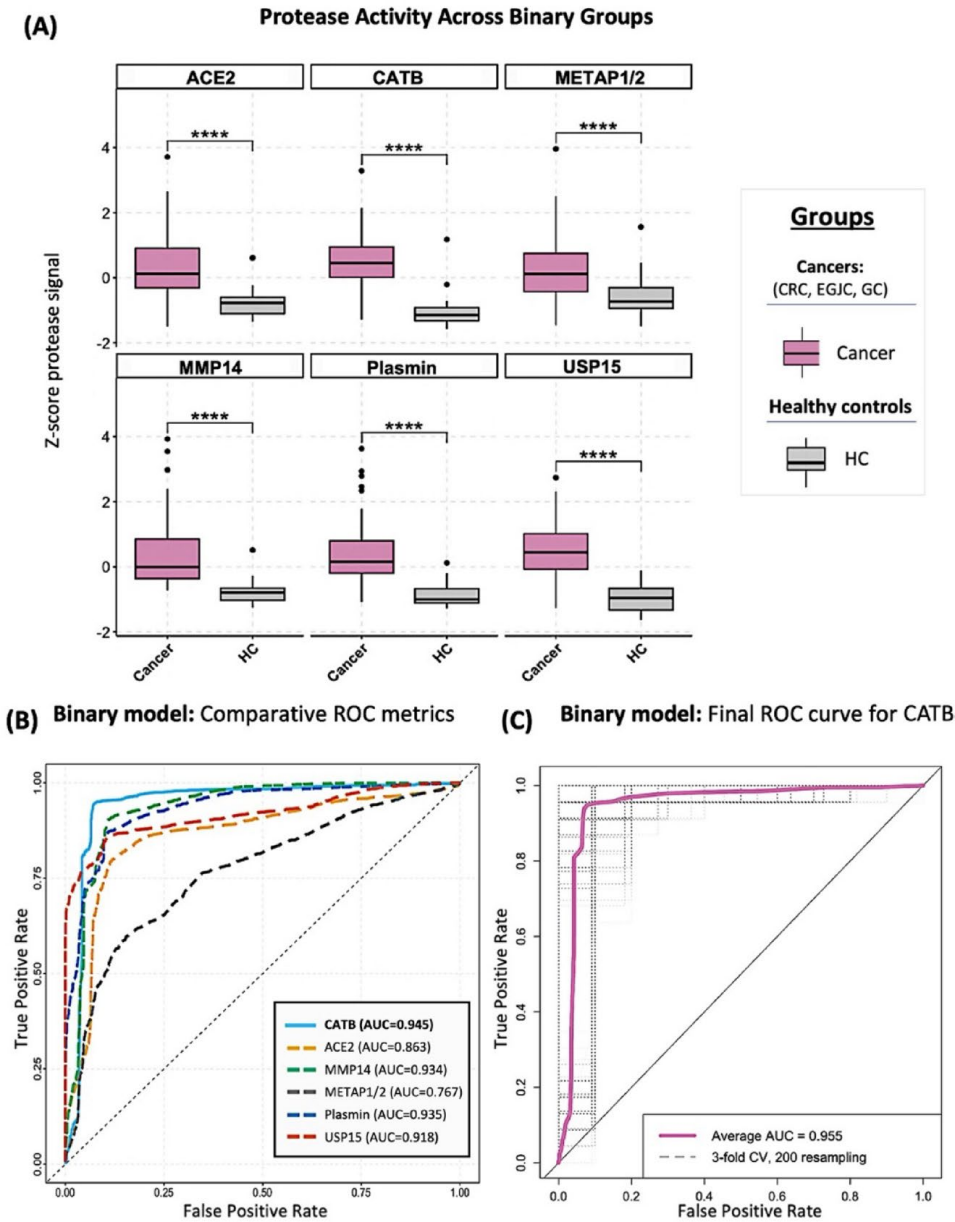
Protease activity profiles and model performance for binary classification of gastrointestinal cancer patients and healthy controls. (**A**) Boxplots showing z-score–normalized protease activity measured using six CCP-derived probes across two clinical groups: healthy controls (HC; *N* = 31) and gastrointestinal cancer patients (*N* = 68; pooled colorectal cancer (CRC), esophagogastric junction cancer (EGJC), and gastric cancer (GC)). Statistical differences were assessed using Kruskal–Wallis tests with Bonferroni correction (*p* < 0.05), followed by post hoc Dunn’s tests for pairwise comparisons: ****p < 0.0001. (**B**) ROC curves comparing AUC values from six binary logistic regression models, each using a single probe as the predictor. CATB demonstrated the highest discriminative performance (AUC = 0.945) based on 3-fold cross-validation with 50 random repeats. (**C**) Final model validation for the CATB-based classifier using 3-fold cross-validation repeated 200 times. The model achieved a mean AUC of 0.955 (SD = 0.045), with mean sensitivity of 0.933 (SD = 0.074) and specificity of 0.906 (SD = 0.060). Gray dashed lines represent AUCs from individual cross-validation folds, highlighting model robustness.

To evaluate the discriminatory power of CCP-derived protease activity profiles across GI cancers, we performed a binary classification analysis comparing pooled GI cancer cases (CRC, EGJC, and GC; *N* = 68) versus HC (*N* = 31). Z-score normalized signals from six individual probes revealed significantly elevated protease activity in the cancer group across all markers (Fig. 9A). Kruskal–Wallis testing with Bonferroni correction confirmed statistically significant differences for all probes (*p* < 0.0001), which were further supported by post hoc Dunn’s tests (Fig. 9A). Among them, CATB exhibited the largest effect size and the most distinct separation between cancer and control samples.

To assess diagnostic utility, six binary logistic regression models were independently trained using each protease probe as a single predictor. ROC curve analysis (Fig. 9B) demonstrated that the CATB-based model yielded the highest classification performance (AUC = 0.945), followed closely by plasmin (AUC = 0.935), MMP14 (AUC = 0.934), and USP15 (AUC = 0.918). In contrast, METAP1/2 showed the lowest performance (AUC = 0.767). These findings identified CATB as the most effective individual biomarker for distinguishing GI cancer patients from healthy individuals. The CATB model was then validated using 3-fold cross-validation with 200 iterations. As shown in Fig. 9C, the final model achieved a robust mean AUC of 0.955 (SD = 0.045), with sensitivity of 0.933 (SD = 0.074) and specificity of 0.906 (SD = 0.060), confirming its stability and clinical potential.

Altogether, we developed binary LR models to classify patients into three clinical groups: CRC, UGIC (EGJC + GC), and HC. Two initial models were constructed to distinguish (1) UGIC versus HC and (2) CRC versus HC. Permutation-based variable importance analysis identified USP15 and plasmin as the most informative predictors for the UGIC (30.3%) and CRC (9.2%) models, respectively (Fig. 8A and B). Based on these findings, single-probe logistic regression classifiers were developed using USP15 for UGIC and plasmin for CRC. Both models demonstrated excellent diagnostic performance, achieving AUCs > 0.97, with sensitivity and specificity exceeding 91% and 90%, respectively (Fig. 8C).

To evaluate the performance of a unified model for pan-GI cancer detection, an additional binary LR model was developed using the CATB probe to classify pooled GI cancers (CRC, EGJC, GC) versus HC. CATB outperformed other markers and yielded an AUC of 0.955, with mean sensitivity of 0.933 and specificity of 0.906 (Fig. 9). All models incorporated a rigorous pipeline, including 3-fold nested cross-validation with random repeats, Firth LR to reduce bias in small-sample inference, and bootstrapping to evaluate coefficient stability.

Table 2 summarizes the optimal predictors and corresponding model performance for each binary classification task. Collectively, these findings highlight USP15, plasmin, and CATB as promising single-marker classifiers for UGIC, CRC, and pooled GI cancer detection, respectively. Future validation in larger, independent cohorts will be essential to assess their translational utility in clinical protease-based diagnostics.

**Table 2:** Summary of binary logistic regression models, optimal predictors, and corresponding figures.

Original datasets (*n*)	Cancer group	Control group	Selected optimal probe	Corresponding figure(s)
Upper GI cancers (UGIC)	EGJC + GC	HC	USP15	Fig. 8C
Lower GI cancer	CRC	HC	Plasmin	
All GI cancers	CRC, EGJC + GC	HC	CATB	Fig. 9B and C

## Discussion

This study presents a noninvasive strategy for detecting and classifying GI cancers through protease activity profiling in plasma using a six-probe CCP panel. By integrating protease-specific activity measurements with LR modeling, we achieved accurate classification of CRC, UGIC, and pooled GI cancer cases versus healthy controls. Our findings demonstrate that individual proteases—USP15, plasmin, and CATB—serve as strong subtype-specific biomarkers capable of distinguishing cancer patients from non-cancer individuals with high accuracy.

Proteases play essential roles in cancer biology, contributing to tumor invasion, metastasis, and microenvironment remodeling^12^. Their elevated activity can be detected in circulation, making them attractive biomarkers for early cancer detection^11^. The CCP assay used in this study capitalizes on this principle by measuring shifts in peptide charge upon proteolytic cleavage, enabling activity-based detection through gel electrophoresis^16^. This approach requires only small plasma volumes and offers a cost-effective, gel-based method for measuring protease activity directly from clinical samples^16^.

The use of peptide-based CCP probes offers several advantages. Peptides are highly specific, allowing for targeted detection of protease activity, which enhances the accuracy of the assay^33,34^. Owing to their small size and modifiable nature, they are ideal for designing probes that can undergo charge changes upon cleavage by specific proteases, enabling easy detection^33,35^. Additionally, peptides are relatively cost-effective and can be synthesized with high purity, making them practical for protease profiling in cancer diagnostics^36,37^.

Initial validation of the CCP probe targeting plasmin confirmed the platform’s specificity and sensitivity, with signal intensity correlating with protease concentration and minimal cross-reactivity with other peptides. This specificity laid the foundation for applying the CCP panel to patient-derived plasma samples. Across all six probes—targeting ACE2, CATB, METAP1/2, MMP14, plasmin, and USP15—significantly elevated protease activity was observed in cancer patients compared to healthy controls. Notably, the CATB probe displayed the most distinct signal separation between cancer and control groups, supporting its potential as a universal GI cancer marker.

Our results align with and extend prior research showing elevated levels of these proteases in cancer patients^16,38–43^. Cathepsin B protease, which our CATB probe can specifically measure, has been widely implicated in tumor invasion and metastasis, and its elevation in our cancer cohorts is consistent with its known biological roles^44^. Similarly, plasmin is a serine protease involved in fibrinolysis and extracellular matrix remodeling, both of which are critical to cancer progression^21^. Ubiquitin specific protease 15, which our USP15 probe can measure specifically, has been shown to regulate multiple oncogenic signaling pathways and modulate immune responses in the tumor microenvironment^45^. The ability to measure these proteases in plasma adds translational value by enabling real-time, systemic monitoring of tumor-associated proteolytic activity.

When examining cancer types, distinct protease activity signatures emerged. CRC samples showed higher activity for plasmin, while UGIC (EGJC + GC) cases were characterized by elevated USP15 activity. These findings were supported by statistical comparisons (*p* < 0.05) and further confirmed by LR modeling. Permutation-based feature importance analysis identified USP15 and Plasmin as the top-ranked predictors for UGIC and CRC models, respectively. Each model achieved strong diagnostic performance, with AUC values exceeding 0.97 and both sensitivity and specificity above 90%.

Beyond subtype classification, we developed a model to distinguish all GI cancer patients from healthy individuals. Among the six probes, CATB provided the best discriminative performance, with an average AUC of 0.955, sensitivity of 0.933, and specificity of 0.906. Importantly, the CATB-based model maintained robustness across 200 random repeats of cross-validation, underscoring its reliability and potential as a single-probe screening tool for GI malignancies. The comparable performance of these single-probe models also demonstrates the feasibility of simplified assays for clinical use, avoiding the complexity and cost of multiplexed biomarker panels.

While MS offers broad proteomic coverage and high analytical specificity, it is resource-intensive—requiring sophisticated instrumentation, trained personnel, and considerable per-sample costs—limiting its accessibility for routine clinical use. ELISA is more cost-effective and widely used but depends on antibody availability and primarily measures protein abundance rather than enzymatic activity, which may not fully reflect disease-associated proteolytic dynamics. In contrast, the CCP assay requires only small volumes of plasma, minimal sample preparation (no albumin depletion), and basic laboratory equipment (e.g., standard gel electrophoresis). It directly measures protease activity, providing functional information, and demonstrates low per-sample cost. These features enhance its potential for scalability and application in resource-limited or high-throughput settings. We also reference our development of the PAC-MANN platform, which adapts the CCP assay for high-throughput implementation, helping to address throughput limitations inherent in gel-based workflows^16^. Furthermore, fluorescence resonance energy transfer (FRET)-based assays often require albumin depletion and substantial plasma dilution to reduce autofluorescence and background interference. In contrast, the CCP assay functions effectively with native plasma, without the need for pre-processing steps such as albumin depletion or dilution. Additionally, CCP probes operate via a charge-shift mechanism that enables detection using standard gel electrophoresis, eliminating the need for specialized fluorescence optics or kinetic readout systems. This reduces background signal and improves assay reproducibility. CCP probes are also structurally simple, cost-effective to synthesize, and exhibit high stability in biological fluids.

Despite the promising outcomes, this study has several limitations. The sample size was modest, particularly within subtypes, which may affect generalizability. Additionally, the healthy control group was not age-matched to the cancer cohorts, which could introduce confounding factors related to age-associated protease activity. Although we employed rigorous cross-validation to mitigate overfitting, validation in larger, independent cohorts will be essential. Additionally, while single-probe models offer simplicity, integrating multiple proteases or clinical features could enhance diagnostic accuracy. Transitioning the CCP assay to high-throughput or automated platforms will also be necessary for routine clinical use, such as the recently developed PAC-MANN assay^16^. Although prior MS validation confirmed specific cleavage for these CCP probes in pancreatic cancer^16^, MS characterization of all CCP probes in other GI cancers remains a valuable goal for future work. Future studies should aim to expand sample diversity, include additional cancer types and stages, and explore integration of the CCP assay into longitudinal monitoring for treatment response or disease recurrence. Evaluating the prognostic value of circulating protease activity could further broaden the clinical applications of this platform.

In conclusion, our study introduces a protease activity profiling platform using CCPs for accurate, noninvasive classification of GI cancers. The identification of USP15, plasmin, and CATB as high-performing biomarkers highlights the potential of activity-based signatures for diagnostic applications. The simplicity, sensitivity, and interpretability of this approach provide a strong foundation for future development of blood-based cancer screening tools. With further validation, the CCP assay could serve as an accessible diagnostic solution for detection and classification of GI cancers.

## Conclusion

This study demonstrates the potential of plasma-based protease activity profiling as a minimally invasive approach for the detection and classification of GI cancers. Using a six-probe CCP panel, we identified distinct enzymatic activity signatures that accurately distinguished CRC, UGIC, and HC. LR models developed with individual probes—plasmin, USP15, and CATB—achieved excellent classification performance, with AUC values exceeding 0.95 and high sensitivity and specificity. The CCP platform, which requires only small plasma volumes and utilizes a simple gel-based electrophoretic readout, provides a cost-effective and scalable strategy for noninvasive cancer screening and subtype differentiation. Further validation in larger, independent cohorts will be critical to confirm these findings and support clinical translation of this activity-based diagnostic platform.

## Supplementary Information

Below is the link to the electronic supplementary material.

Supplementary Material 1

Supplementary Material 2

Supplementary Material 3

## Data Availability

The data generated during and/or analyzed during the current study are available from the corresponding author upon request.
