# Supplementary material for: Profiling plasma protease activity with charge-changing peptides enables detection and classification of gastrointestinal cancers
Source: Sci Rep. 2025 Sep 1;15:32184. doi: 10.1038/s41598-025-17915-0 (PMC12402143; doi:10.1038/s41598-025-17915-0)
Supplement: Supplementary file 1 — Supplementary Material 1 [file 41598_2025_17915_MOESM1_ESM.pdf]

HPLC ANALYSIS REPORT

Sample: ACE2 Analyzed date: 2022-7-23

Sequence: Ac-GEPEPFAGAGK-NH2

Lot. No.: P67884-220713

Column: 250*4.6mm,Kromasil-C18-5um

Solvent A A: 0.1% Trifluoroacetic Acid in 100% Acetonitrile

Solvent B B: 0.1% Trifluoroacetic Acid in 100% Water

Gradient:

	A	B
0.0min	18%	82%
25.0min	43%	57%
25.1min	100%	0%
30.0min	Stop	

Volume: 10µl

Wavelength: 220nm

Flow rate: 1.0ml/min

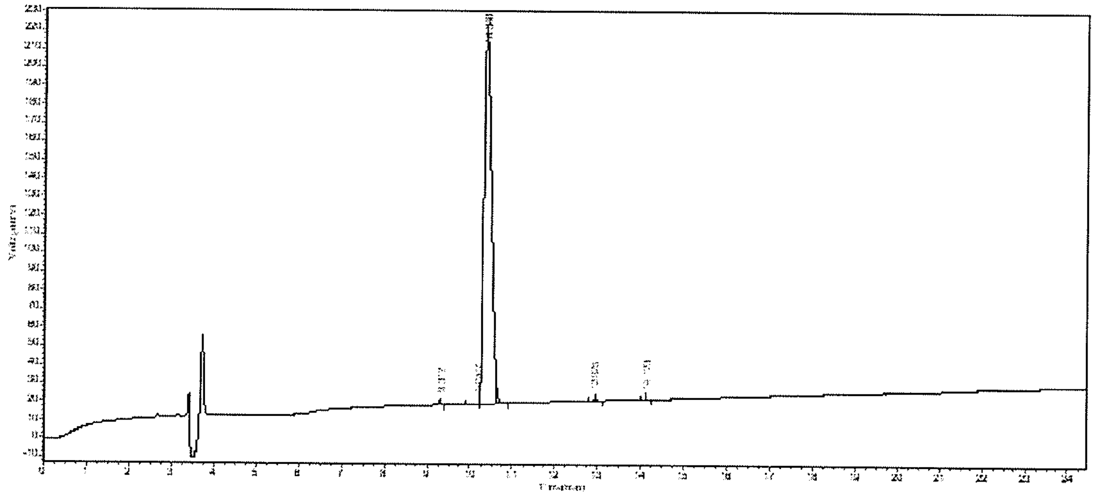

Peak No	Ret Time	Height	Area	Conc.
1	9.312	30.572	153.254	0.0062
2	10.207	400.451	1846.367	0.0742
3	10.348	200531.484	2449693.500	98.5119
4	10.348	11526.740	29395.725	1.1821
5	12.925	334.968	2462.855	0.0990
6	14.123	361.101	3146.488	0.1265
Total				100.00

 Copyright © Bio Basic Inc. All rights reserved.

BIO BASIC® – Your Supplier and Manufacturer of Life Science Products and Services

MS ANALYSIS REPORT

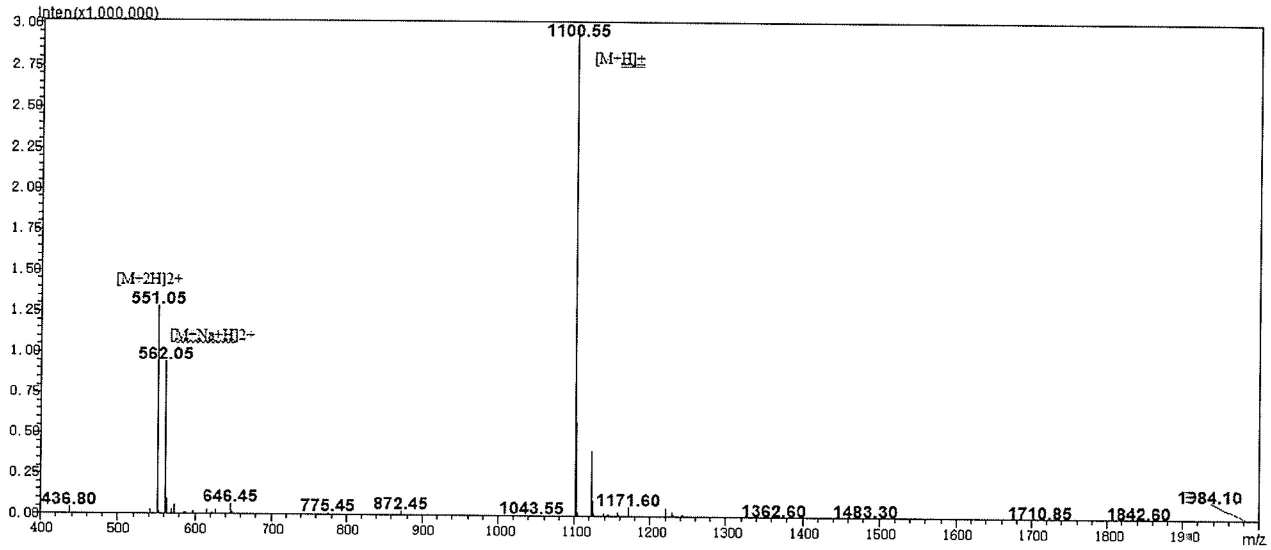

Sample Description

Analyzed date:	2022/7/22	Instrument	SHIMADZU LCMS-2020		
		Probe:	ESI	Probe Bias:	+4.5kv
		Nebulizer Gas Flow:	1.5L/min	Detector:	1.2kv
Sample:	ACE2	CDL:	-20.0v	T. Flow:	0.2ml/min
M.W.:	1100.18	CDL Temp.:	250 °C	B. Conc.:	50%H2O/50%ACN
Lot. No.:	P67884-220713	Block Temp.:	400 °C		

 Copyright © Bio Basic Inc. All rights reserved.

HPLC ANALYSIS REPORT

Sample: CATB Analyzed date: 2022-7-21

Sequence: Ac-DGLAGGAGGK-NH2

Lot. No.: P67887-220713

Column: 4.6×250mm,ChromCore 120 C18 5u

Solvent A A: 0.1% Trifluoroacetic Acid in 100% Acetonitrile

Solvent B B: 0.1% Trifluoroacetic Acid in 100% Water

Gradient:

	A	B
0.0min	10%	90%
25.0min	35%	65%
25.1min	100%	0%
30.0min	Stop	

Volume: 10μl

Wavelength: 220nm

Flow rate: 1.0ml/min

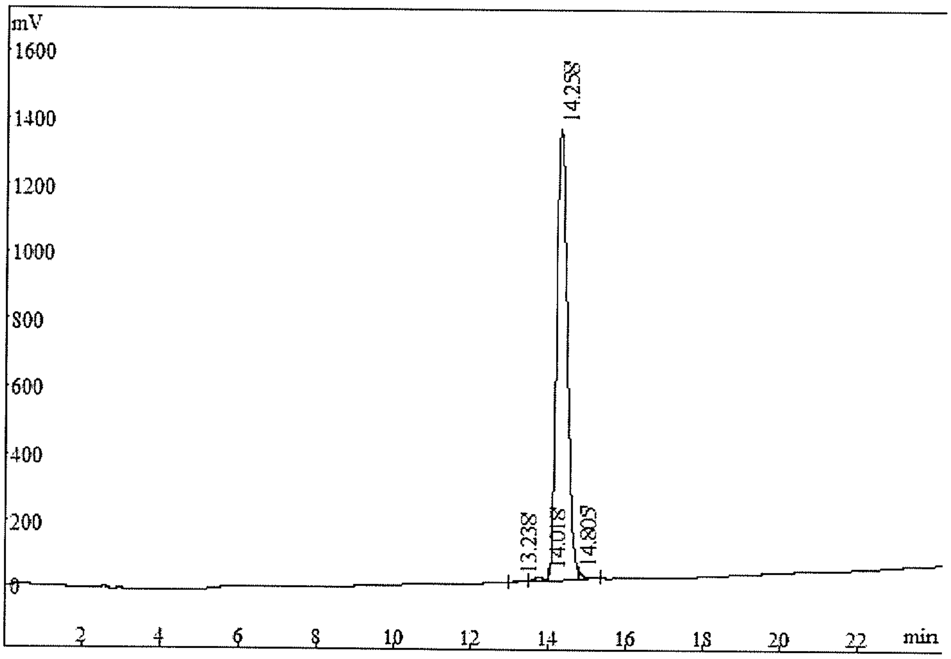

Rank	Time	Conc.	Area	Height
1	13.238	0.1337	33308	1929
2	14.018	0.7202	179356	15701
3	14.258	98.34	24491054	1345619
4	14.805	0.804	200221	18245
Total		100	24903939	1381494

 Copyright © Bio Basic Inc. All rights reserved.

MS ANALYSIS REPORT

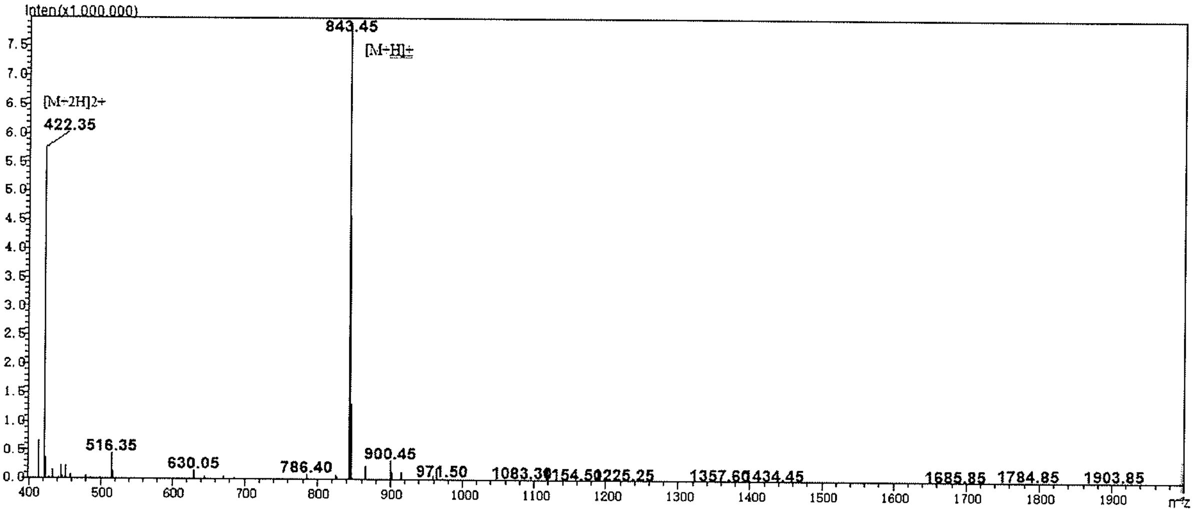

Sample Description		Instrument	SHIMADZU LCMS-2020		
Analyzed date:	2022/7/20	Probe:	ESI	Probe Bias:	+4.5kv
		Nebulizer Gas Flow:	1.5L/min	Detector:	1.2kv
Sample:	CATB	CDL:	-20.0v	T. Flow:	0.2ml/min
M.W.:	842.89	CDL Temp.:	250 °C	B. Conc.:	50%H2O/50%ACN
Lot. No.:	P67887-220713	Block Temp.:	400 °C		

 Copyright © Bio Basic Inc. All rights reserved.

HPLC ANALYSIS REPORT

Sample: METAP1/2 Analyzed date: 2022-7-24
Sequence: Ac-DGDGMARTLK-NH2
Lot. No.: P67886-220713
Column: 250*4.6mm,Kromasil-C18-5um
Solvent A A: 0.1% Trifluoroacetic Acid in 100% Acetonitrile
Solvent B B: 0.1% Trifluoroacetic Acid in 100% Water
Gradient:

	A	B
0.0min	16%	84%
25.0min	41%	59%
25.1min	100%	0%
30.0min	Stop	

Volume: 10μl
Wavelength: 220nm
Flow rate: 1.0ml/min

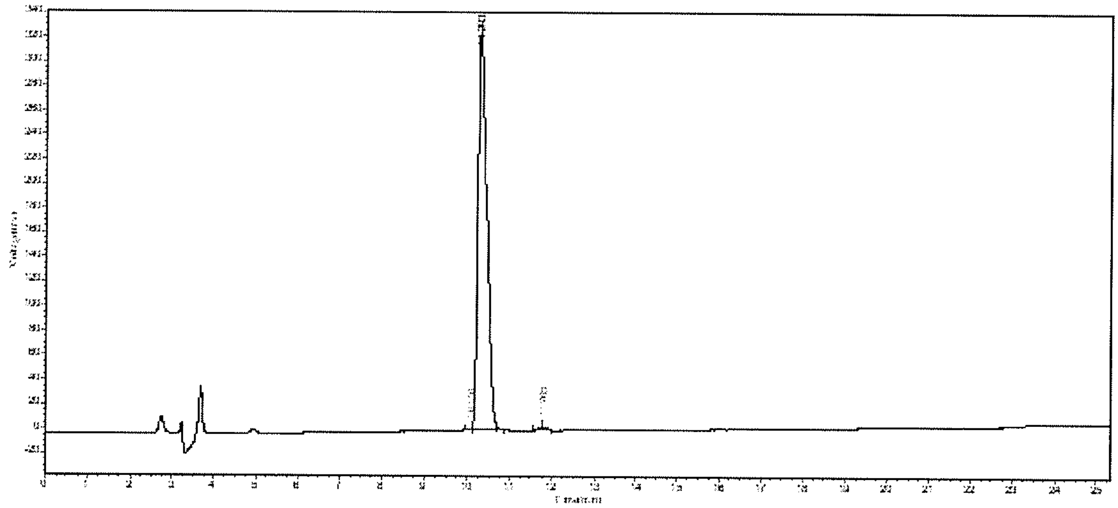

Peak No	Ret Time	Height	Area	Conc.
1	10.110	1299.190	5168.042	0.1136
2	10.293	324818.219	4501521.500	98.9073
3	10.293	2631.652	7401.694	0.1626
4	11.765	2627.570	37162.199	0.8165
Total				100.00

BIO BASIC® – Your Supplier and Manufacturer of Life Science Products and Services

MS ANALYSIS REPORT

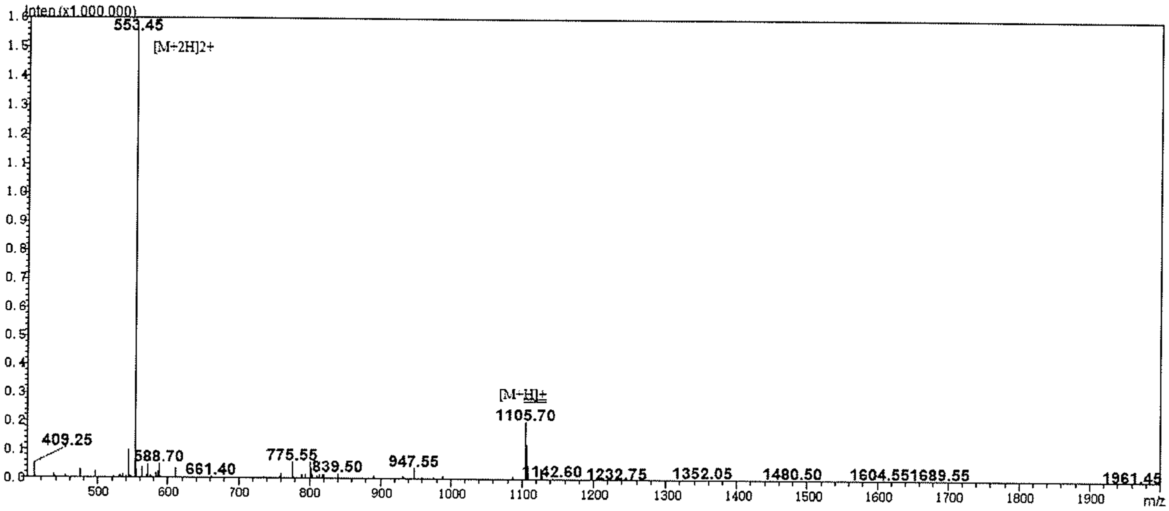

Sample Description		Instrument	SHIMADZU LCMS-2020		
Analyzed date:	2022/7/23	Probe:	ESI	Probe Bias:	+4.5kv
		Nebulizer Gas Flow:	1.5L/min	Detector:	1.2kv
Sample:	METAP1/2	CDL:	-20.0v	T. Flow:	0.2ml/min
M.W.:	1104.23	CDL Temp.:	250 °C	B. Conc.:	50%H2O/50%ACN
Lot. No.:	P67886-220713	Block Temp.:	400 °C		

 Copyright © Bio Basic Inc. All rights reserved.

BIO BASIC® – Your Supplier and Manufacturer of Life Science Products and Services

HPLC ANALYSIS REPORT

Sample:	MMP14	Analyzed date:	2022-7-23
Sequence:	Ac-DGDPAGLRGAGK-NH2		
Lot. No.:	P67890-220713		
Column:	250*4.6mm,Kromasil-C18-5um		
Solvent A	A: 0.1% Trifluoroacetic Acid in 100% Acetonitrile		
Solvent B	B: 0.1% Trifluoroacetic Acid in 100% Water		

Gradient:

	A	B
0.0min	12%	88%
25.0min	37%	63%
25.1min	100%	0%
30.0min	Stop	

Volume: 10µl

Wavelength: 220nm

Flow rate: 1.0ml/min

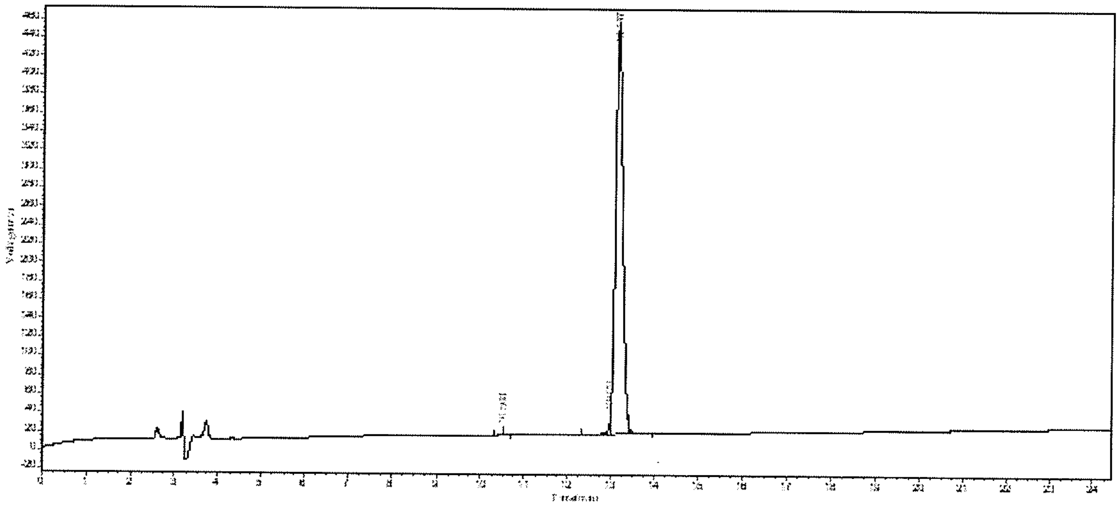

Peak No	Ret Time	Height	Area	Conc.
1	10.508	703.682	6700.934	0.1308
2	12.978	13872.413	36158.359	0.7060
3	13.147	431445.969	5038793.500	98.3835
4	13.147	15310.475	39933.680	0.7797
Total				100.00

Bio Basic Canada Inc. | 20 Konrad Crescent | Markham | Ontario L3R 8T4 | | Bio Basic Inc. | 4160 Bailey Avenue | Amherst | New York | 14226
Tel: 1-(800) 313 7224, (905) 474 4493, Fax: (905) 474 5794, Web: www.biobasic.com, Email: order@biobasic.com, Copyright © Bio Basic Inc. All rights reserved.

BIO BASIC® – Your Supplier and Manufacturer of Life Science Products and Services

MS ANALYSIS REPORT

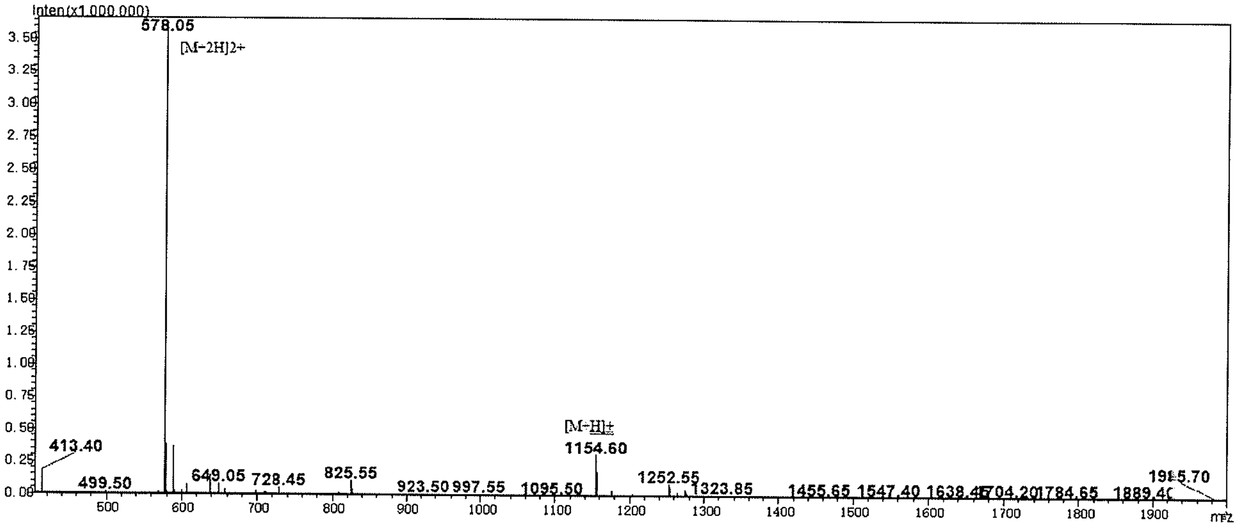

Sample Description		Instrument	SHIMADZU LCMS-2020		
Analyzed date:	2022/7/22	Probe:	ESI	Probe Bias:	+4.5kv
		Nebulizer Gas Flow:	1.5L/min	Detector:	1.2kv
Sample:	MMP14	CDL:	-20.0v	T. Flow:	0.2ml/min
M.W.:	1154.23	CDL Temp.:	250 °C	B. Conc.:	50%H2O/50%ACN
Lot. No.:	P67890-220713	Block Temp.:	400 °C		

 Copyright © Bio Basic Inc. All rights reserved.

HPLC ANALYSIS REPORT

Sample: Plasmin Analyzed date: 2022-7-21
Sequence: Ac-DGDPSLRSVSGK-NH2
Lot. No.: P67888-220713
Column: 4.6×250mm,ChromCore 120 C18 5u
Solvent A A: 0.1% Trifluoroacetic Acid in 100% Acetonitrile
Solvent B B: 0.1% Trifluoroacetic Acid in 100% Water
Gradient:

	A	B
0.0min	13%	87%
25.0min	38%	62%
25.1min	100%	0%
30.0min	Stop	

Volume: 10μl
Wavelength: 220nm
Flow rate: 1.0ml/min

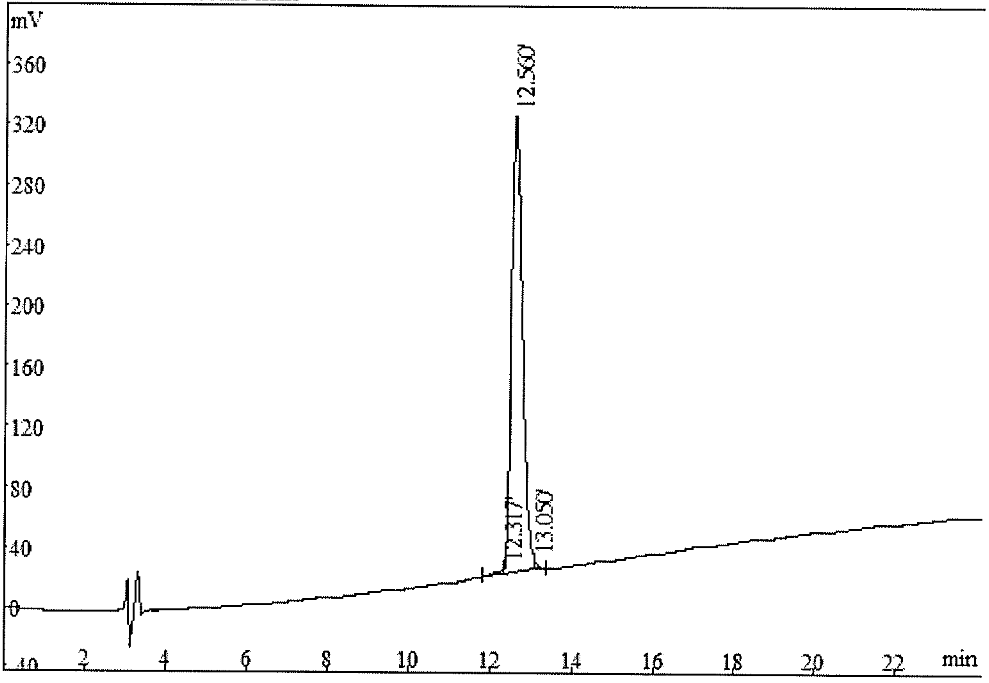

Rank	Time	Conc.	Area	Height
1	12.317	0.7822	42607	3074
2	12.560	98.66	5373728	301692
3	13.050	0.5616	30590	6043
Total		100	5446925	310809

 Copyright © Bio Basic Inc. All rights reserved.

MS ANALYSIS REPORT

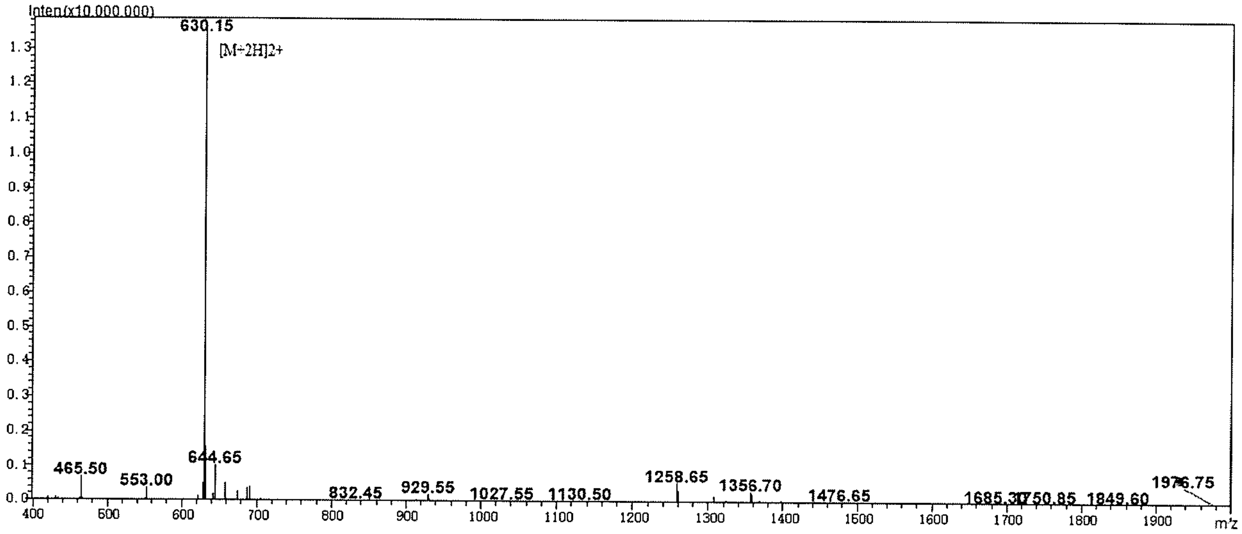

Sample Description

Analyzed date: 2022/7/20

Sample: Plasmin

M.W.: 1258.33

Lot. No.: P67888-220713

Instrument	SHIMADZU LCMS-2020		
Probe:	ESI	Probe Bias:	+4.5kv
Nebulizer Gas Flow:	1.5L/min	Detector:	1.2kv
CDL:	-20.0v	T. Flow:	0.2ml/min
CDL Temp.:	250 °C	B. Conc.:	50%H2O/50%ACN
Block Temp.:	400 °C		

 Copyright © Bio Basic Inc. All rights reserved.

HPLC ANALYSIS REPORT

Sample: USP15 Analyzed date: 2022-7-22

Sequence: Ac-DGDLRGGMPGSGK-NH2

Lot. No.: P67885-220713

Column: 4.6×250mm,ChromCore 120 C18 5u

Solvent A A: 0.1% Trifluoroacetic Acid in 100% Acetonitrile

Solvent B B: 0.1% Trifluoroacetic Acid in 100% Water

Gradient:

	A	B
0.0min	14%	86%
25.0min	39%	61%
25.1min	100%	0%
30.0min	Stop	

Volume: 10μl

Wavelength: 220nm

Flow rate: 1.0ml/min

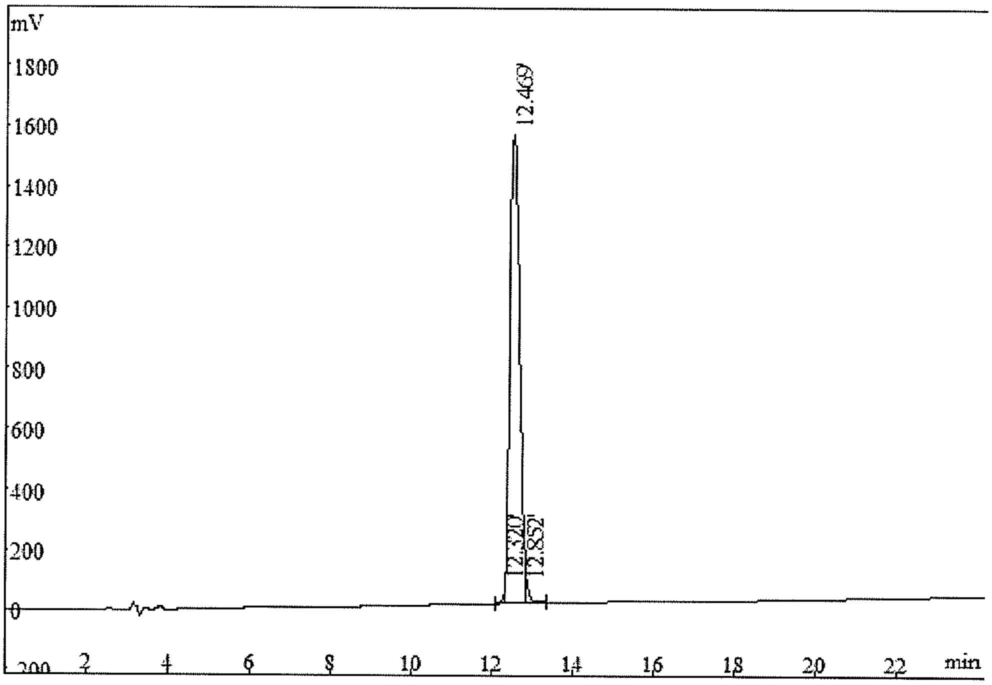

Rank	Time	Conc.	Area	Height
1	12.320	0.6839	173138	56501
2	12.469	98.32	24893781	1544509
3	12.852	0.9924	251254	53272
Total		100	25318173	1654282

 Copyright © Bio Basic Inc. All rights reserved.

MS ANALYSIS REPORT

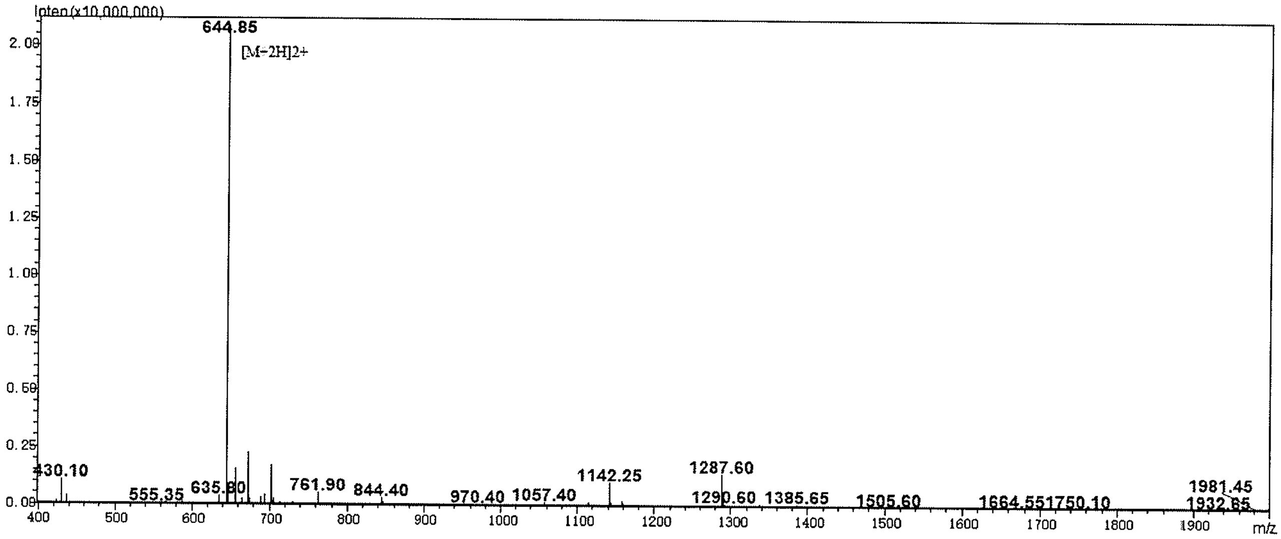

Sample Description

Analyzed date: 2022/7/20

Sample: USP15

M.W.: 1287.40

Lot. No.: P67885-220713

Instrument	SHIMADZU LCMS-2020		
Probe:	ESI	Probe Bias:	+4.5kv
Nebulizer Gas Flow:	1.5L/min	Detector:	1.2kv
CDL:	-20.0v	T. Flow:	0.2ml/min
CDL Temp.:	250 °C	B. Conc.:	50%H2O/50%ACN
Block Temp.:	400 °C		
