# Supplementary material for: Profiling plasma protease activity with charge-changing peptides enables detection and classification of gastrointestinal cancers
Source: Sci Rep. 2025 Sep 1;15:32184. doi: 10.1038/s41598-025-17915-0 (PMC12402143; doi:10.1038/s41598-025-17915-0)

The orginal gel images for CCP assay (Figure 2) (1/2)

Figure 2A (rigth, cropped; left, origin)

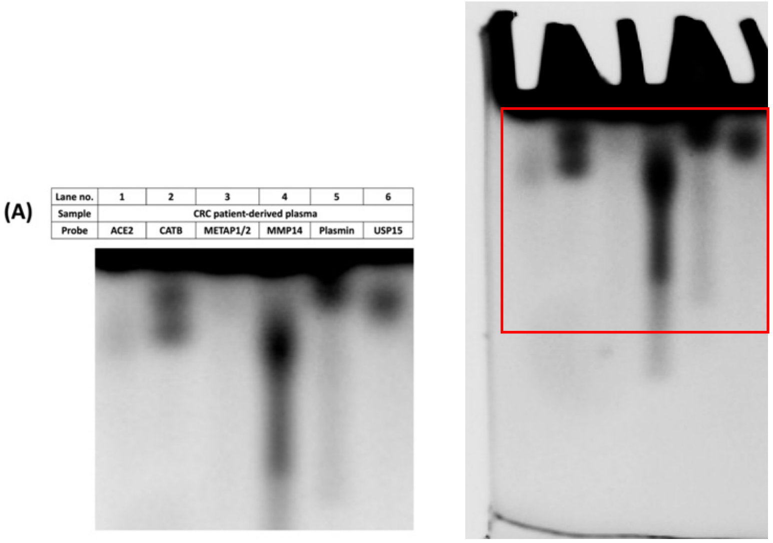

Figure 2B (rigth, cropped; left, origin)

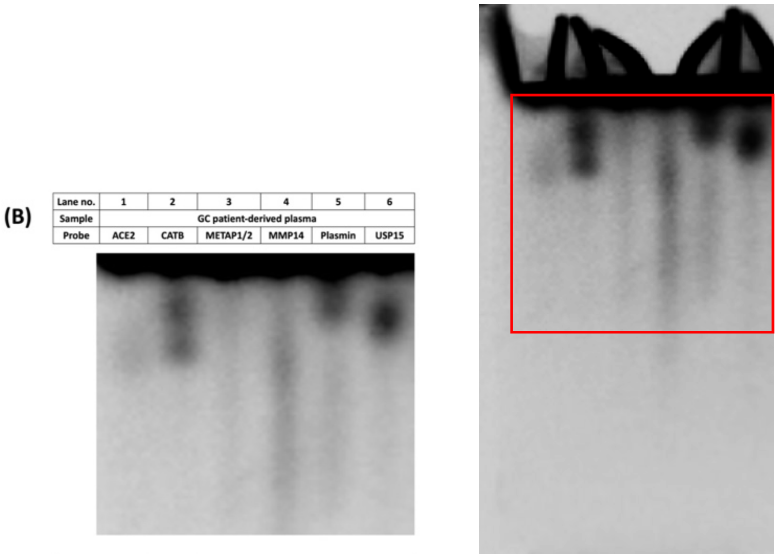

The orginal gel images for CCP assay (Figure 2) (2/2)

Figure 2C (rigth, cropped; left, origin)

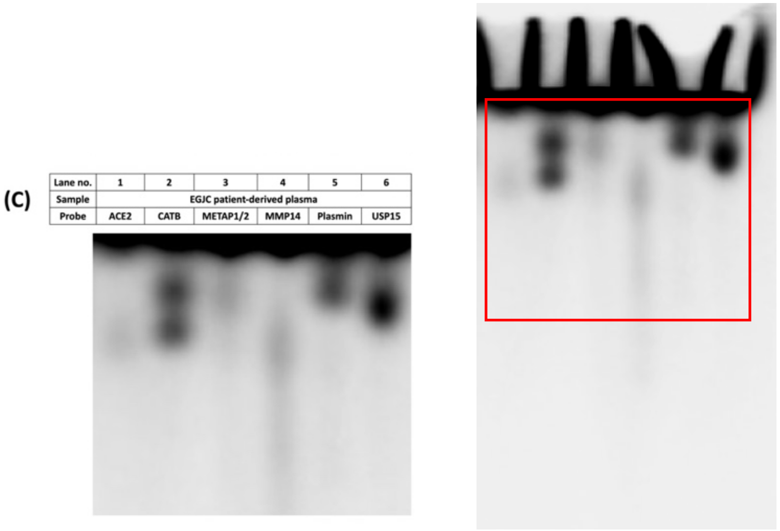

Figure 2D (rigth, cropped; left, origin)

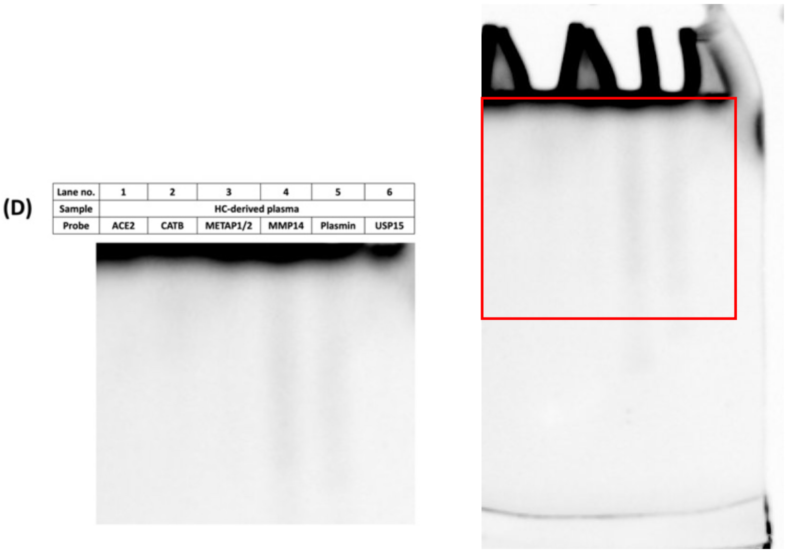

The orginal gel images for selectivity and sensitivity analyses of plasmin probe (Figures S1A and S1C) (1/1)

Figure S1A (rigth, cropped; left, origin)

(A)

Lane no.	1	2	3	4	5	6
Sample	Plasmin (250 nM)					
Probe	ACE2	CATB	METAP1/2	MMP14	Plasmin	USP15

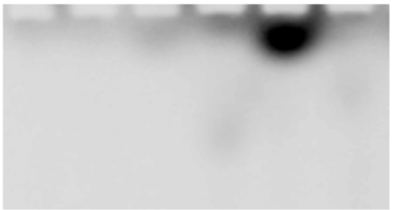

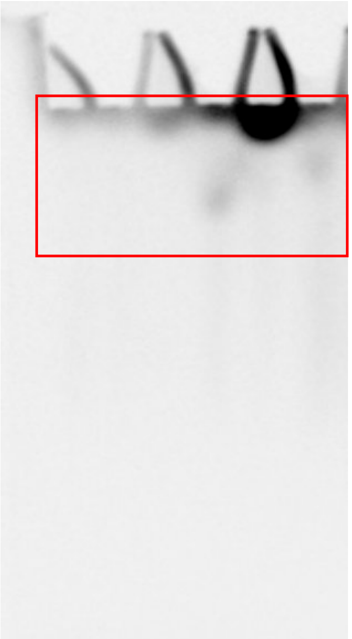

Figure S1C (rigth, cropped; left, origin)

(C)

Lane no.	1	2	3	4	5	6	7	8	9	10	11	12
Plasmin con. (nM)	0	0.98	1.95	3.91	7.81	15.63	31.25	62.5	125	250	500	1000
Probe	Plasmin											

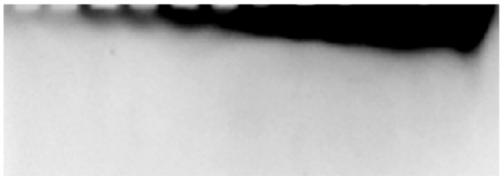

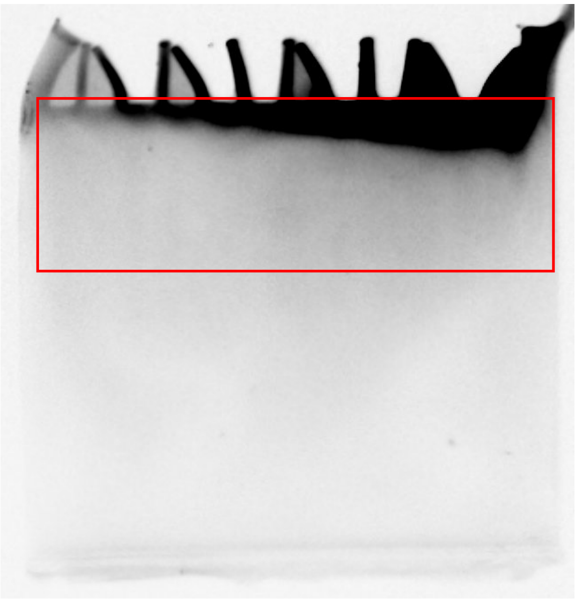

The orginal gel images for CCP assay of CRC group (1/3)

CRC001

ACE2 | CATB | METAP1/2 | MMP14 | Plasmin | USP15

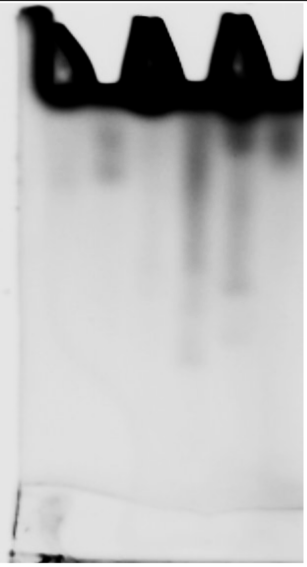

CRC002

ACE2 | CATB | METAP1/2 | MMP14 | Plasmin | USP15

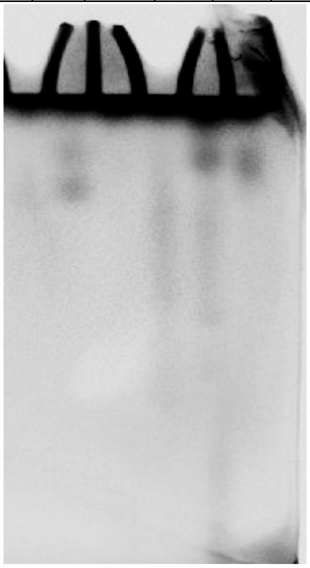

CRC003

ACE2 | CATB | METAP1/2 | MMP14 | Plasmin | USP15

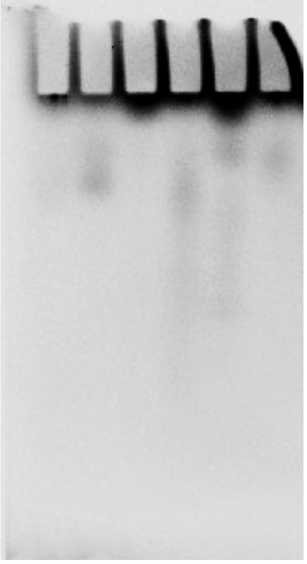

CRC004

ACE2 | CATB | METAP1/2 | MMP14 | Plasmin | USP15

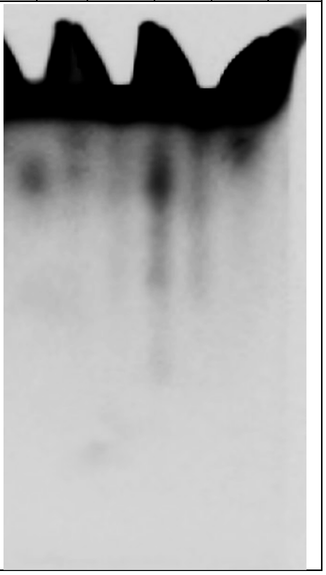

CRC005

ACE2 | CATB | METAP1/2 | MMP14 | Plasmin | USP15

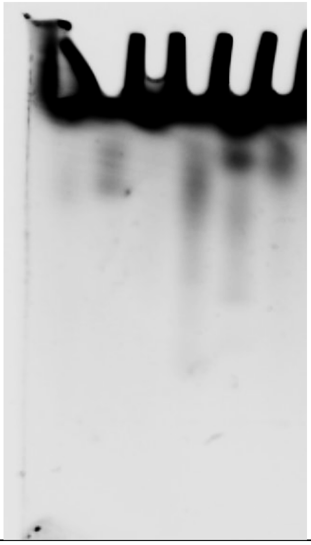

CRC006

ACE2 | CATB | METAP1/2 | MMP14 | Plasmin | USP15

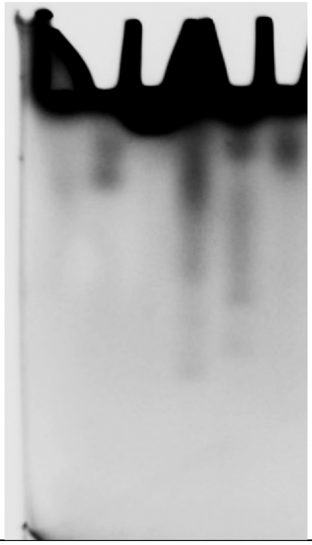

Assay ID: CRC007

ACE2 | CATB | METAP1/2 | MMP14 | Plasmin | USP15

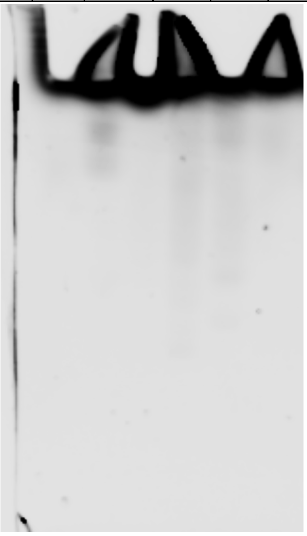

CRC008

ACE2 | CATB | METAP1/2 | MMP14 | Plasmin | USP15

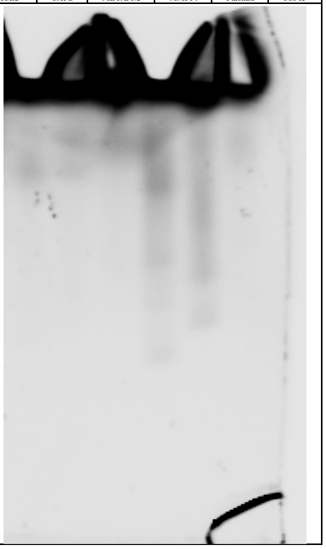

CRC009

ACE2 | CATB | METAP1/2 | MMP14 | Plasmin | USP15

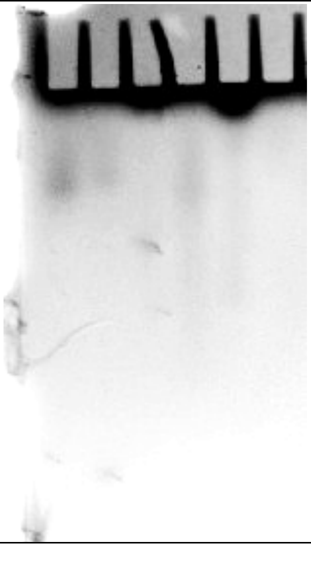

CRC010

ACE2 | CATB | METAP1/2 | MMP14 | Plasmin | USP15

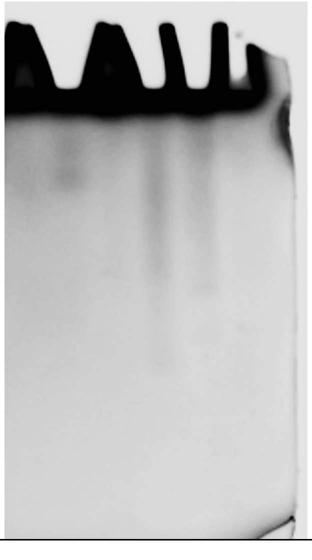

CRC011

ACE2 | CATB | METAP1/2 | MMP14 | Plasmin | USP15

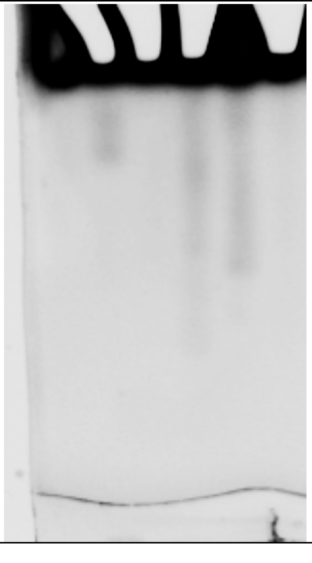

CRC012

ACE2 | CATB | METAP1/2 | MMP14 | Plasmin | USP15

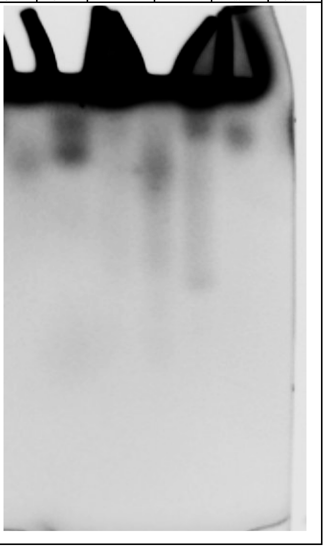

The orginal gel images for CCP assay of CRC group (2/3)

CRC013

ACE2	CATB	METAP1/2	MMP14	Plasmin	USP15

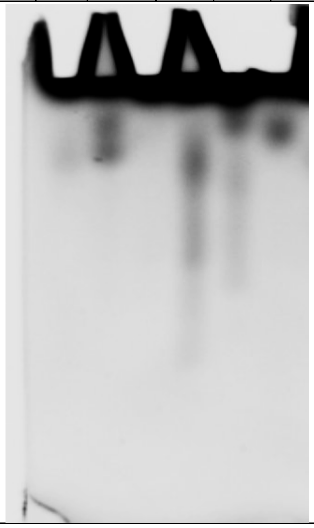

CRC014

ACE2	CATB	METAP1/2	MMP14	Plasmin	USP15

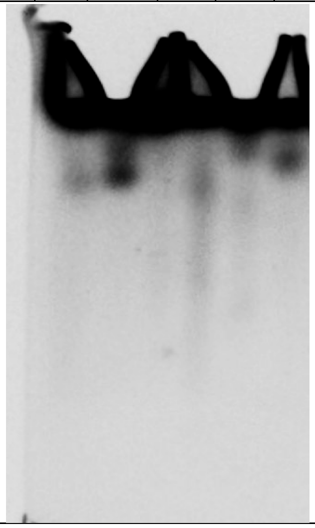

CRC015

ACE2	CATB	METAP1/2	MMP14	Plasmin	USP15

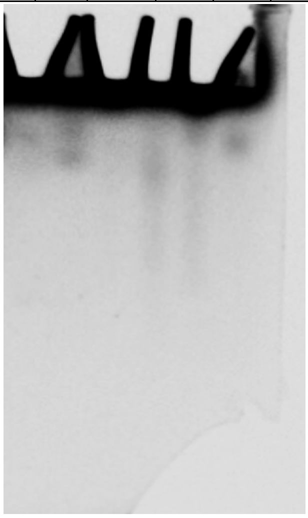

CRC016

ACE2	CATB	METAP1/2	MMP14	Plasmin	USP15

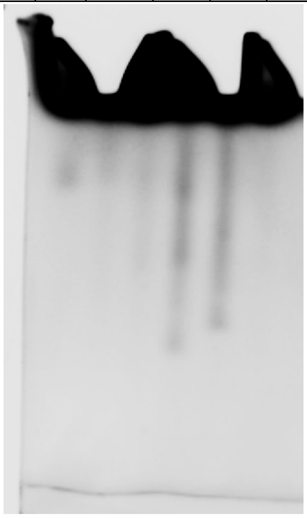

CRC017

ACE2	CATB	METAP1/2	MMP14	Plasmin	USP15

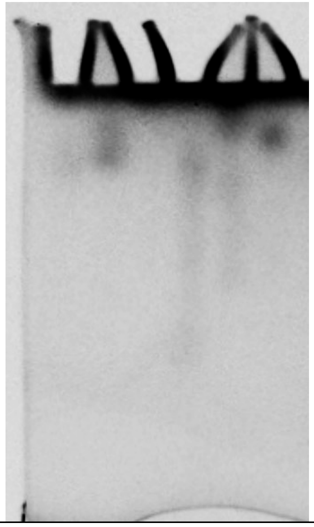

CRC018

ACE2	CATB	METAP1/2	MMP14	Plasmin	USP15

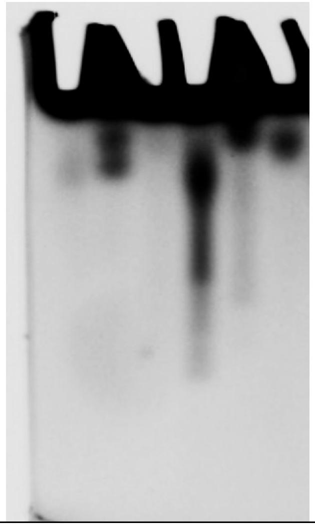

CRC019

ACE2	CATB	METAP1/2	MMP14	Plasmin	USP15

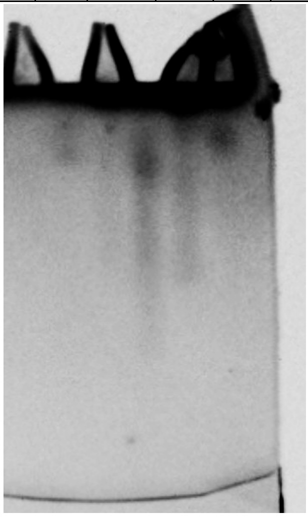

CRC020

ACE2	CATB	METAP1/2	MMP14	Plasmin	USP15

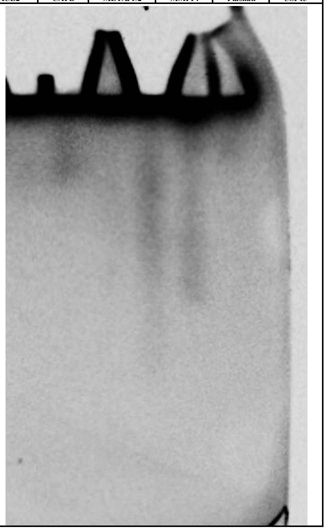

CRC021

ACE2	CATB	METAP1/2	MMP14	Plasmin	USP15

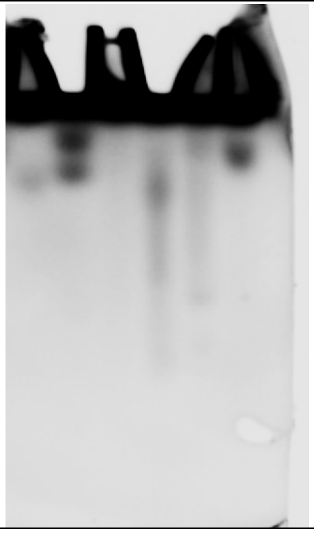

CRC022

ACE2	CATB	METAP1/2	MMP14	Plasmin	USP15

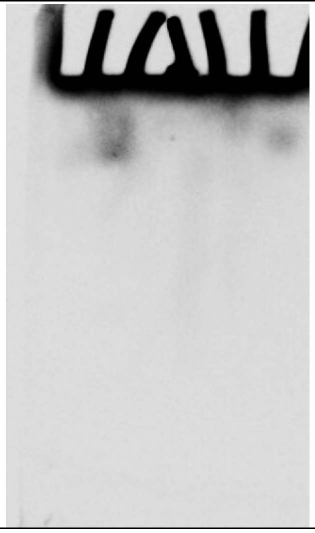

CRC023

ACE2	CATB	METAP1/2	MMP14	Plasmin	USP15

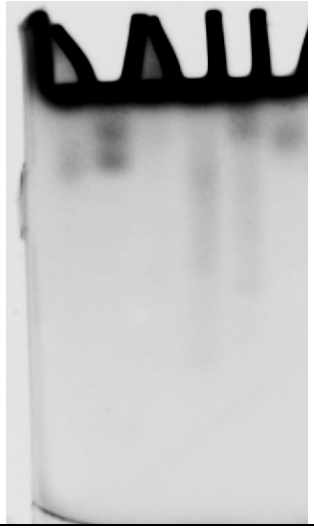

CRC024

ACE2	CATB	METAP1/2	MMP14	Plasmin	USP15

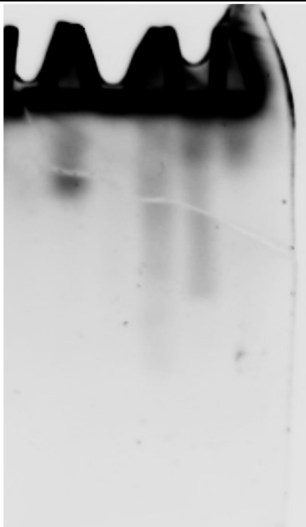

The orginal gel images for CCP assay of CRC group (3/3)

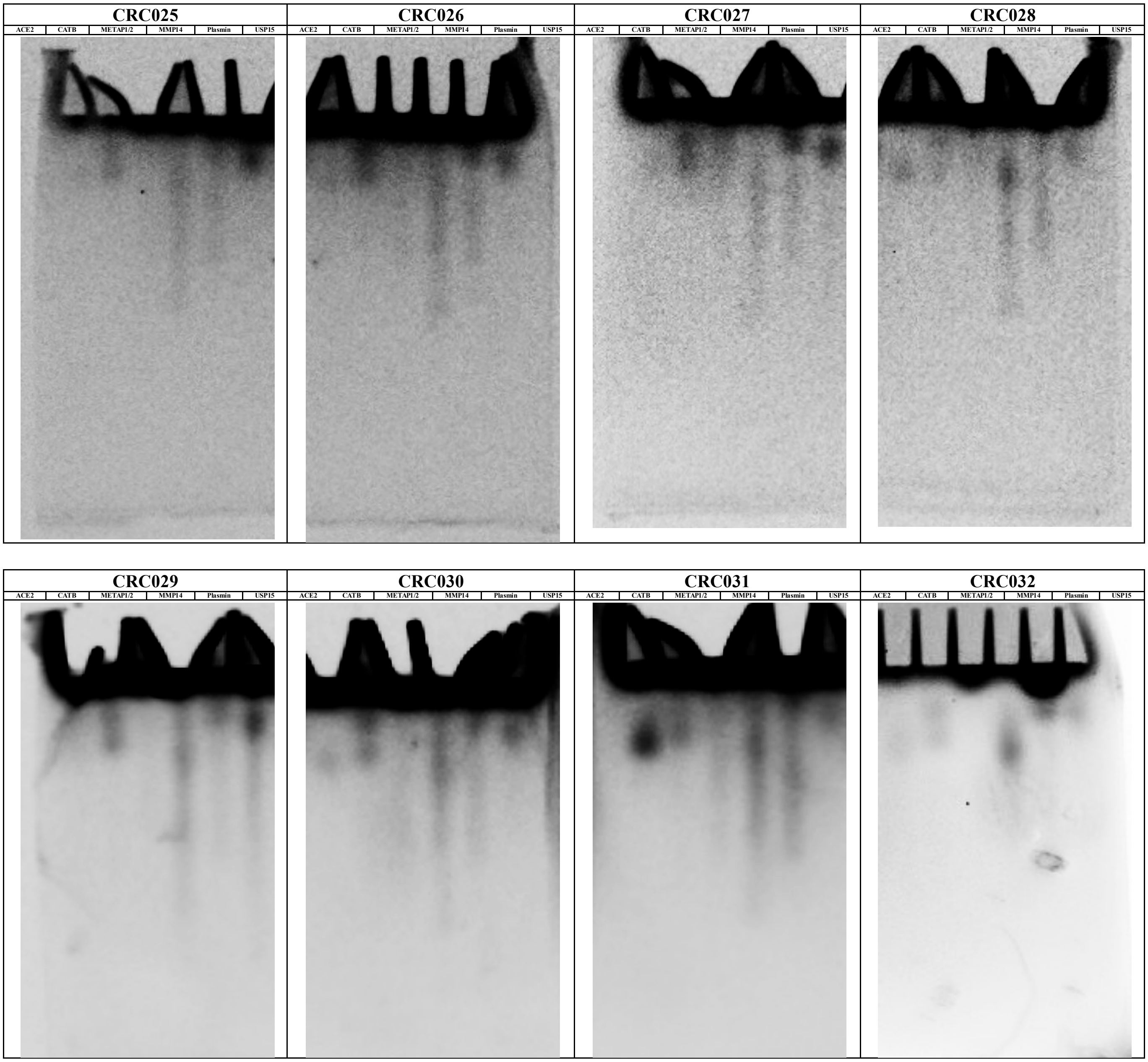

The orginal gel images for CCP assay of GC group (1/2)

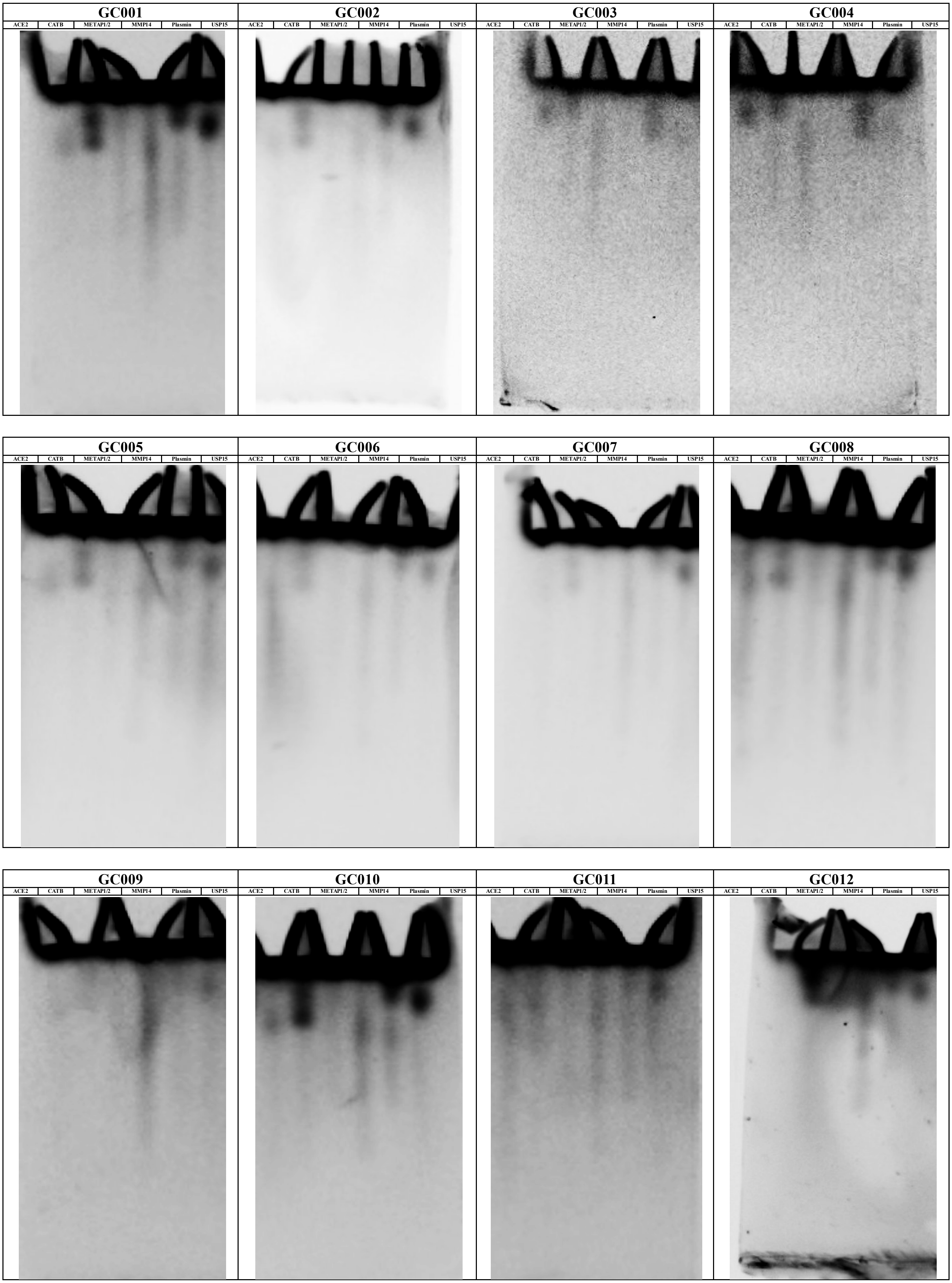

The orginal gel images for CCP assay of GC group (2/2)

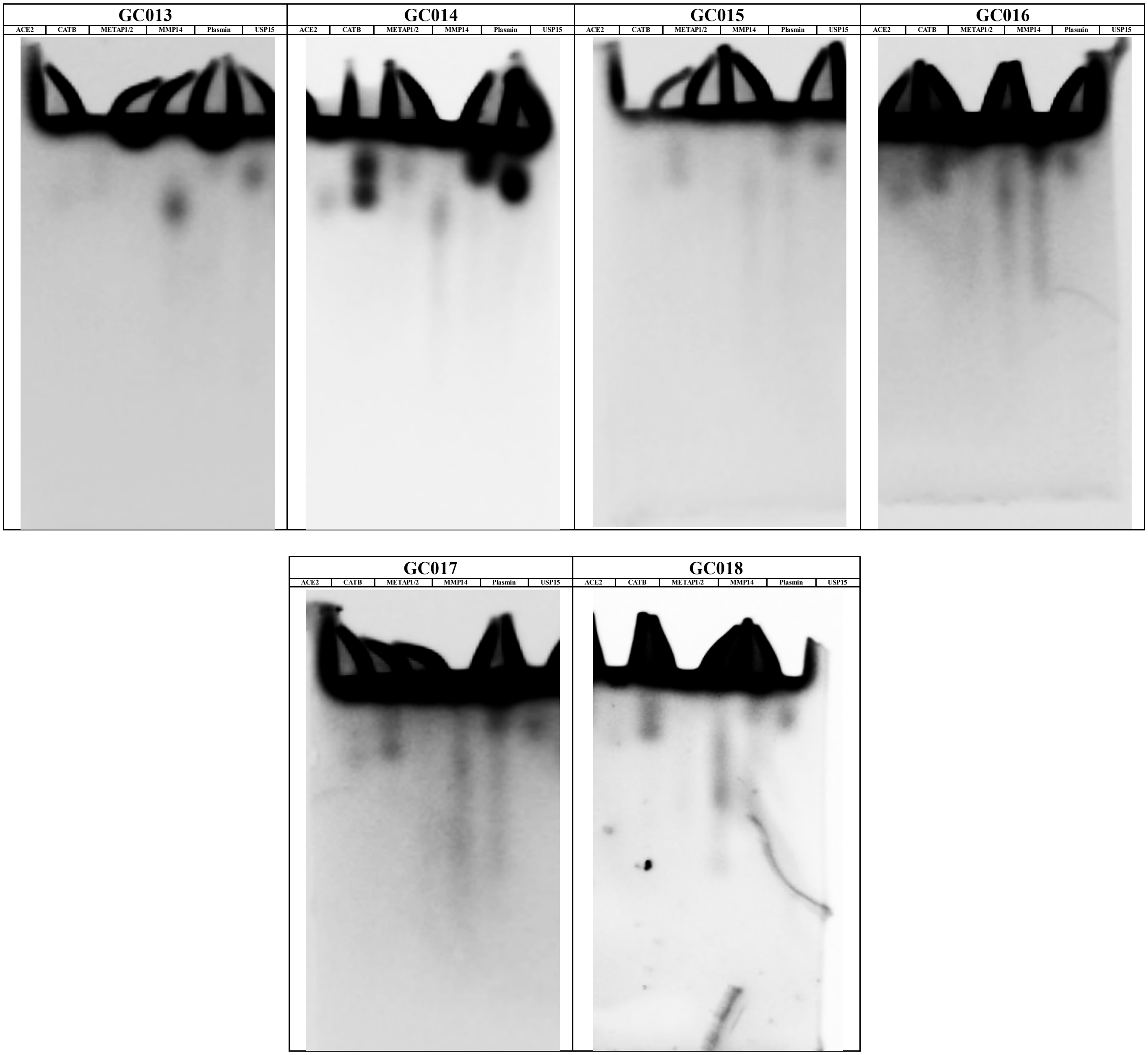

The orginal gel images for CCP assay of EGJC group (1/2)

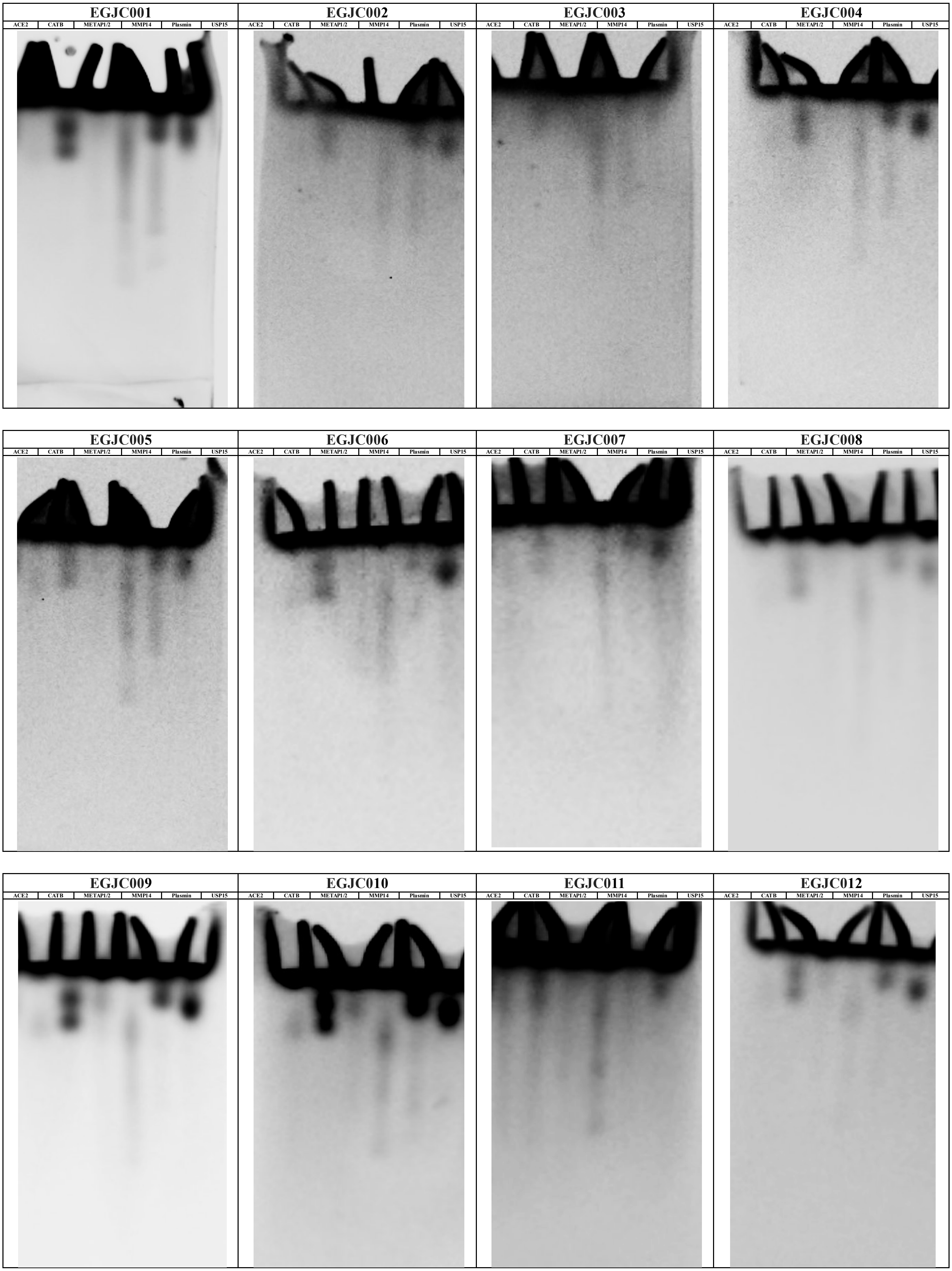

The orginal gel images for CCP assay of EGJC group (2/2)

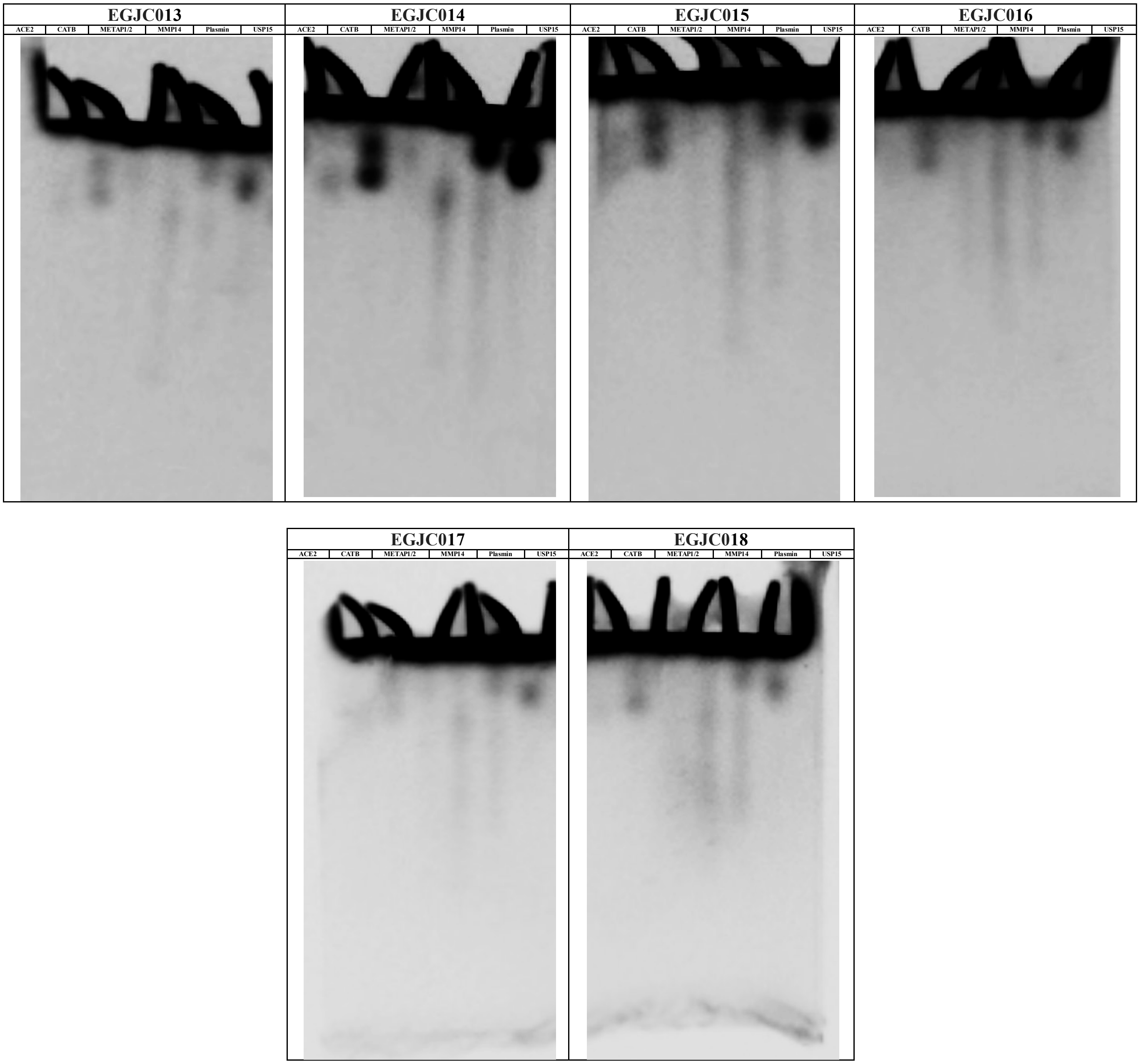

The orginal gel images for CCP assay of HC group (1/3)

HC001

ACE2 | CATB | METAP1/2 | MMP14 | Plasmin | USP15

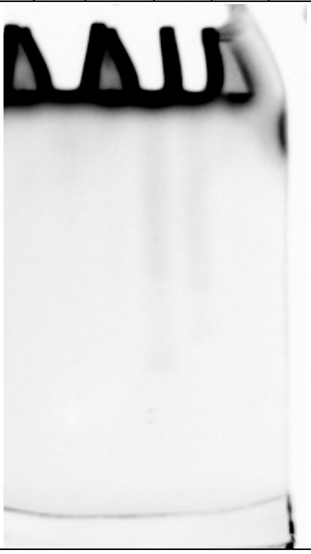

HC002

ACE2 | CATB | METAP1/2 | MMP14 | Plasmin | USP15

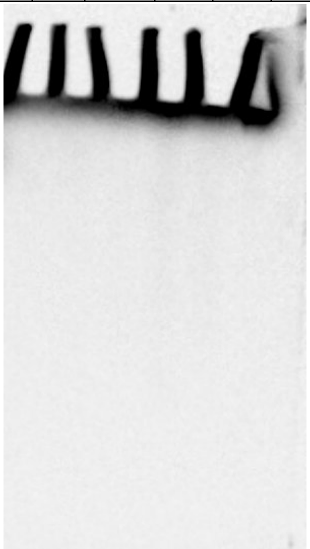

HC003

ACE2 | CATB | METAP1/2 | MMP14 | Plasmin | USP15

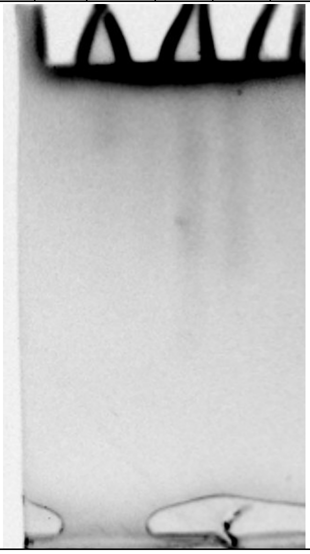

HC004

ACE2 | CATB | METAP1/2 | MMP14 | Plasmin | USP15

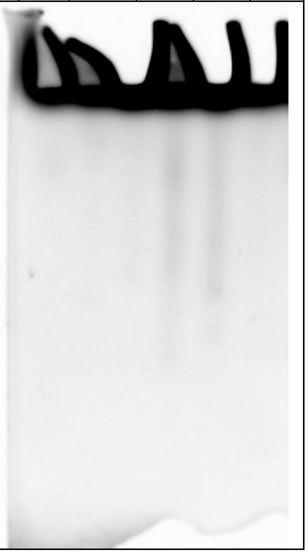

HC005

ACE2 | CATB | METAP1/2 | MMP14 | Plasmin | USP15

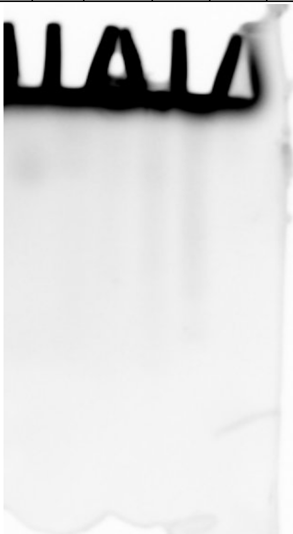

HC006

ACE2 | CATB | METAP1/2 | MMP14 | Plasmin | USP15

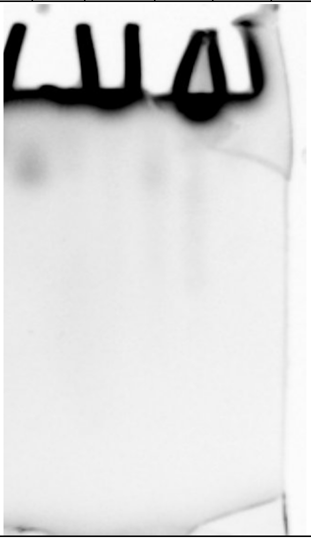

HC007

ACE2 | CATB | METAP1/2 | MMP14 | Plasmin | USP15

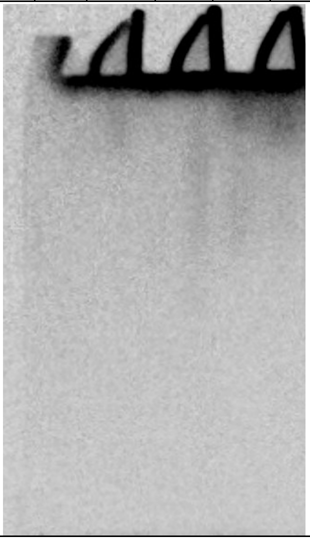

HC008

ACE2 | CATB | METAP1/2 | MMP14 | Plasmin | USP15

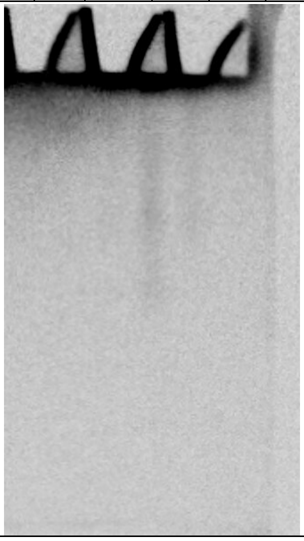

HC009

ACE2 | CATB | METAP1/2 | MMP14 | Plasmin | USP15

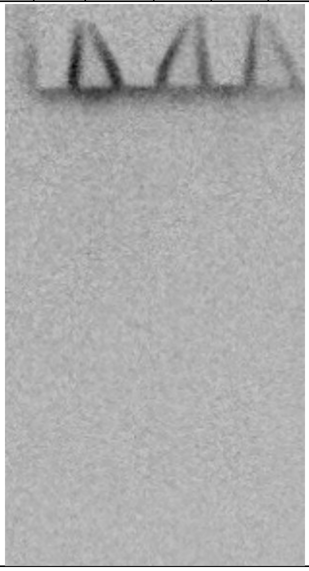

HC010

ACE2 | CATB | METAP1/2 | MMP14 | Plasmin | USP15

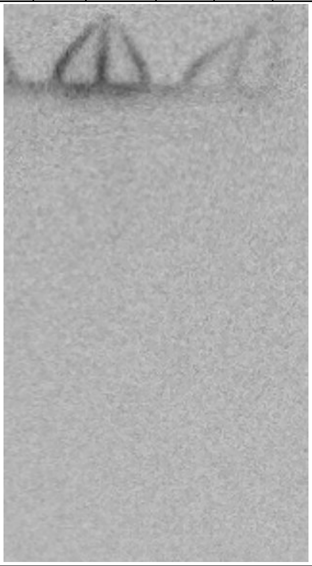

HC011

ACE2 | CATB | METAP1/2 | MMP14 | Plasmin | USP15

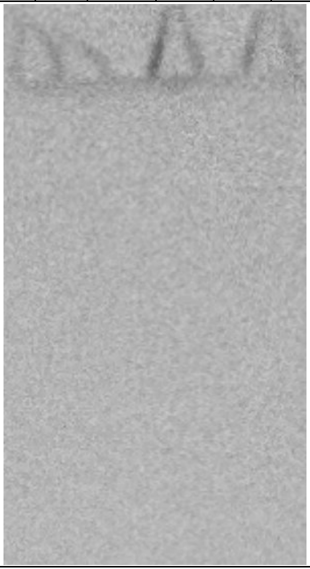

HC012

ACE2 | CATB | METAP1/2 | MMP14 | Plasmin | USP15

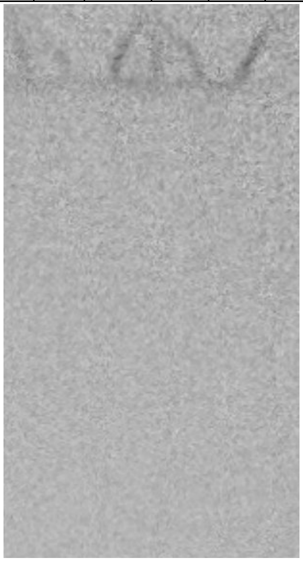

The orginal gel images for CCP assay of HC group (2/3)

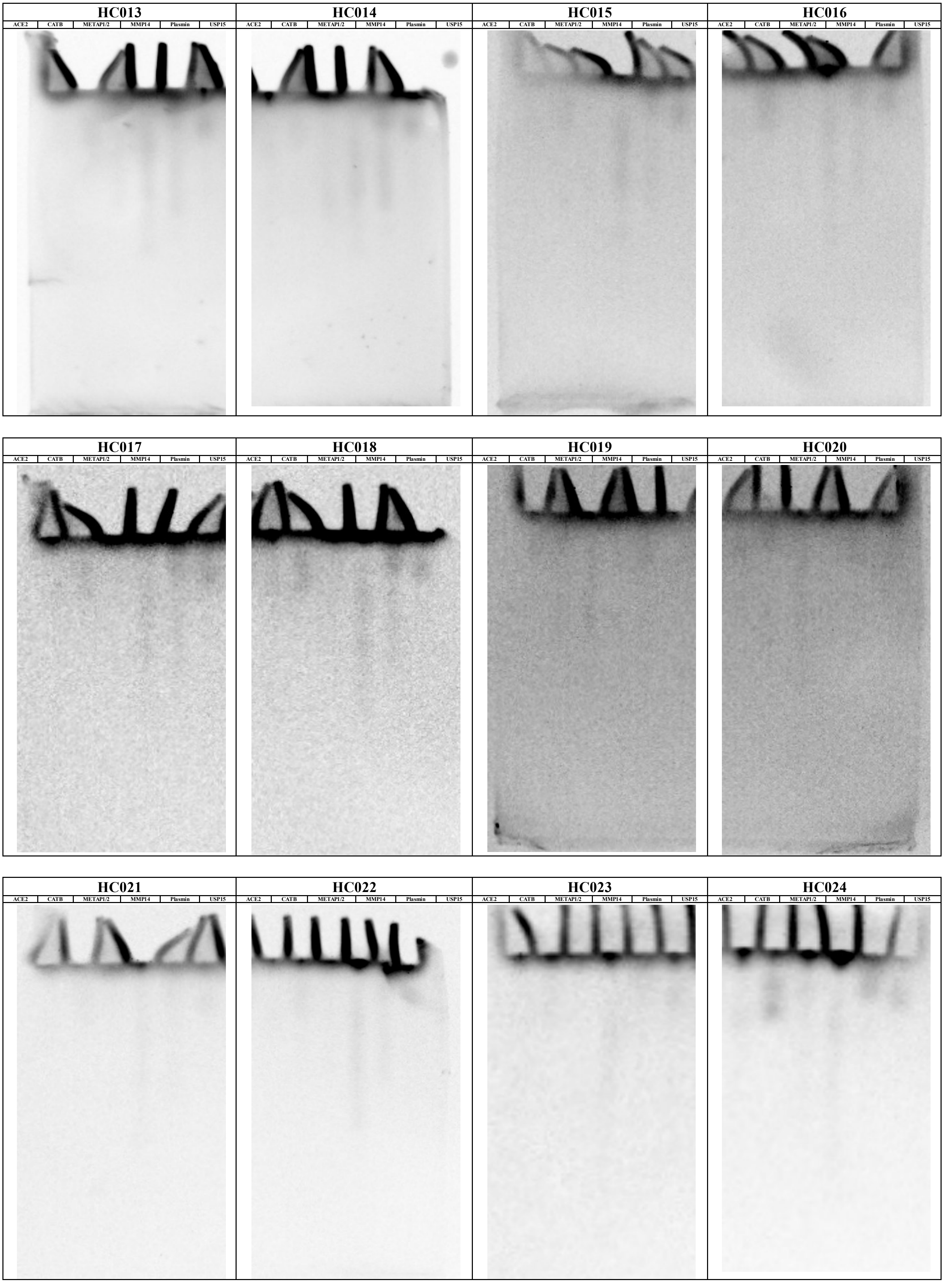

The orginal gel images for CCP assay of HC group (3/3)

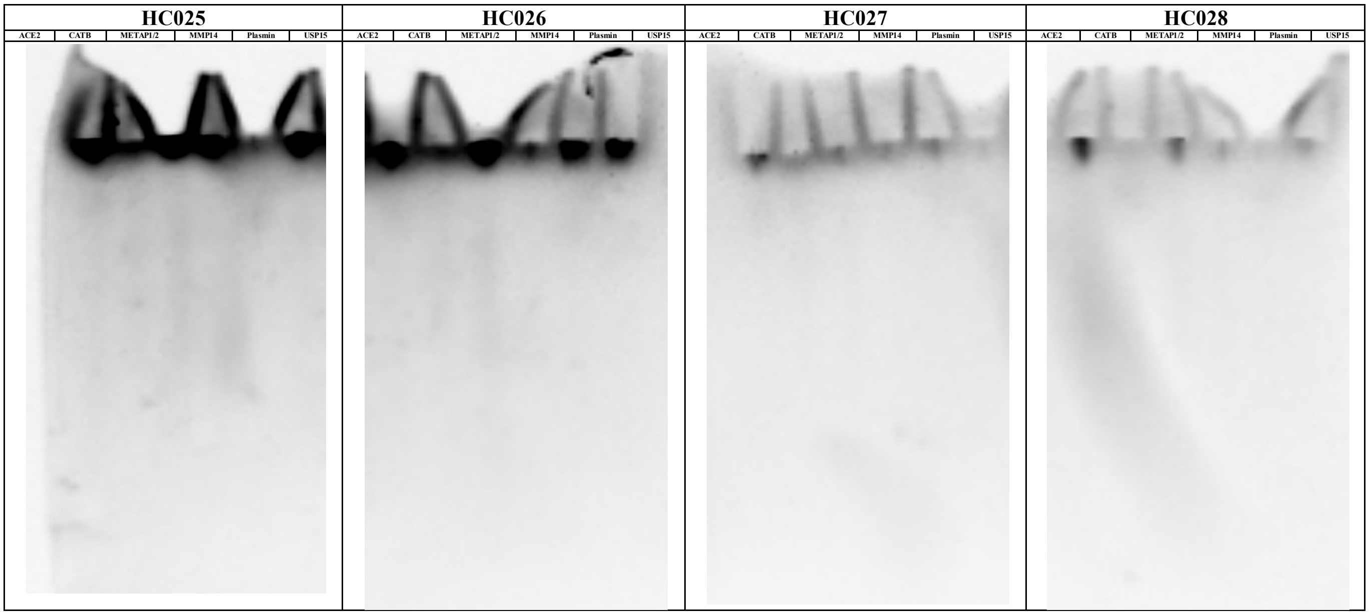

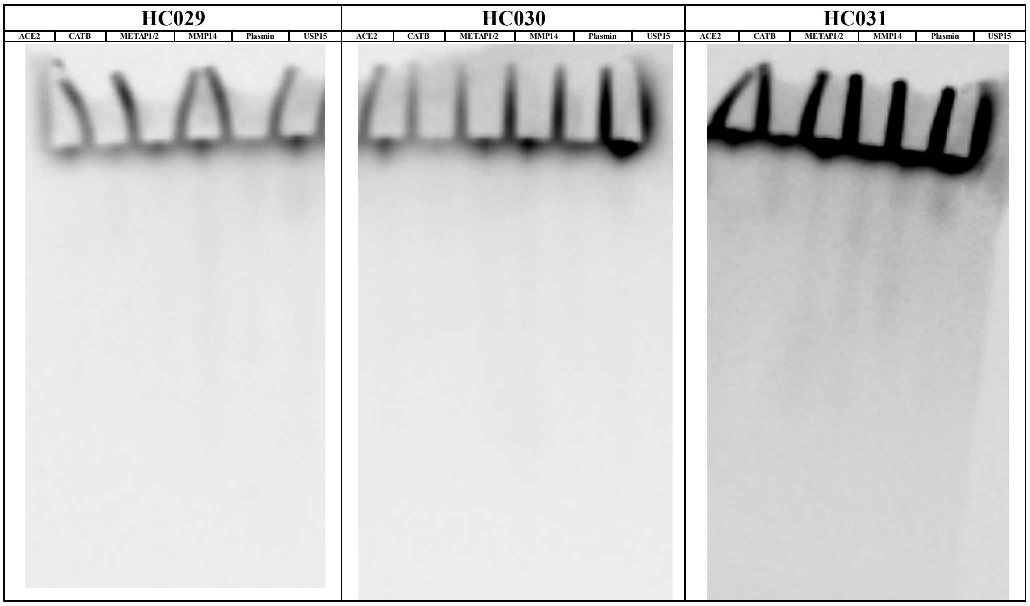

Supplement: Supplementary file 2 — Supplementary Material 2 [file 41598_2025_17915_MOESM2_ESM.pdf]
