# Supplementary material for: Profiling plasma protease activity with charge-changing peptides enables detection and classification of gastrointestinal cancers
Source: Sci Rep. 2025 Sep 1;15:32184. doi: 10.1038/s41598-025-17915-0 (PMC12402143; doi:10.1038/s41598-025-17915-0)
Supplement: Supplementary file 3 — Supplementary Material 3 [file 41598_2025_17915_MOESM3_ESM.pdf]

Supplementary

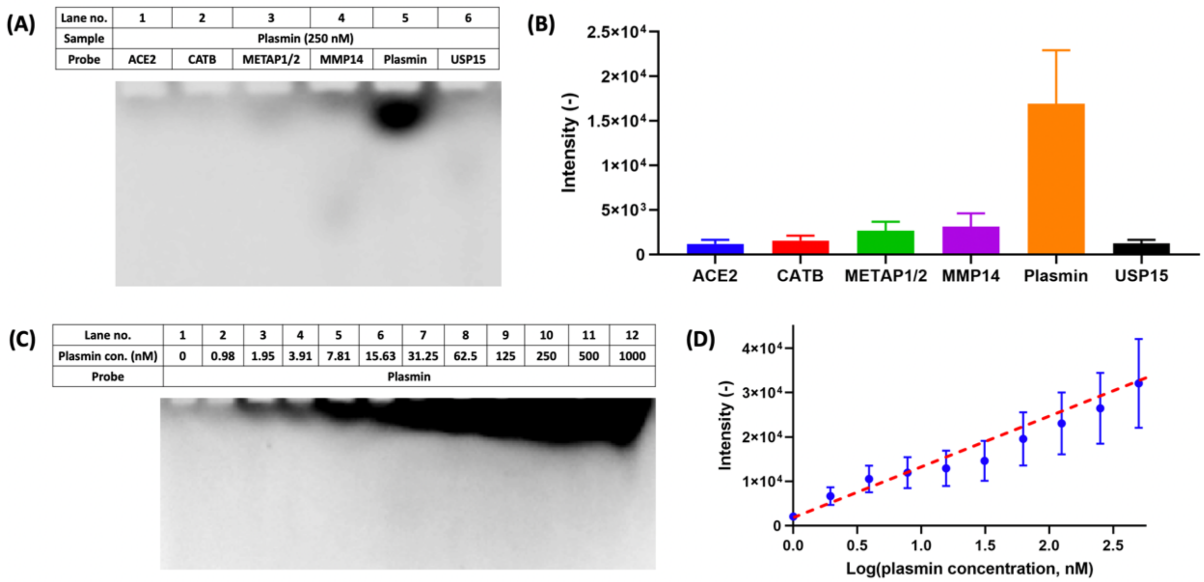

Figure S1: Selectivity and Sensitivity Evaluation of the Charge-Changing Peptide Probe for Plasmin Detection. (A) Gel fluorescence image and (B) corresponding intensity signals demonstrate the charge-changing peptide (CCP) probe's selectivity for plasmin, showing little to no cross-reactivity with other proteases (N=3). (C) Gel fluorescence image and (D) fluorescence intensity highlights the sensitivity of the CCP probe, revealing a positive correlation between signal strength and plasmin concentration (N=3).

Table S1: Demographic Characteristics of Study Participants

	**CRC*** (N=32)	**GC*** (N=18)	**EGJC*** (N=18)	**HC*** (N=31)
**Age**				
Mean ± SD (years)	62 ± 12	62 ± 9	66 ± 10	34 ± 9
**Sex**				
Male	57 %	50 %	73 %	44 %
Female	43 %	50 %	27 %	56 %
**Stage**				
I	-	-	-	-
II	17 %	25 %	-	-
III	43 %	50 %	67 %	-
IV	30 %	25 %	33 %	-
**Pathology grade**				
Well differentiated	-	-	-	-
Moderately differentiated	100 %	25 %	67 %	-
Poorly differentiated	-	75 %	33 %	-

*CRC, colorectal cancer; GC, gastric cancer; EGJC, esophagogastric junction cancer; HC, healthy control

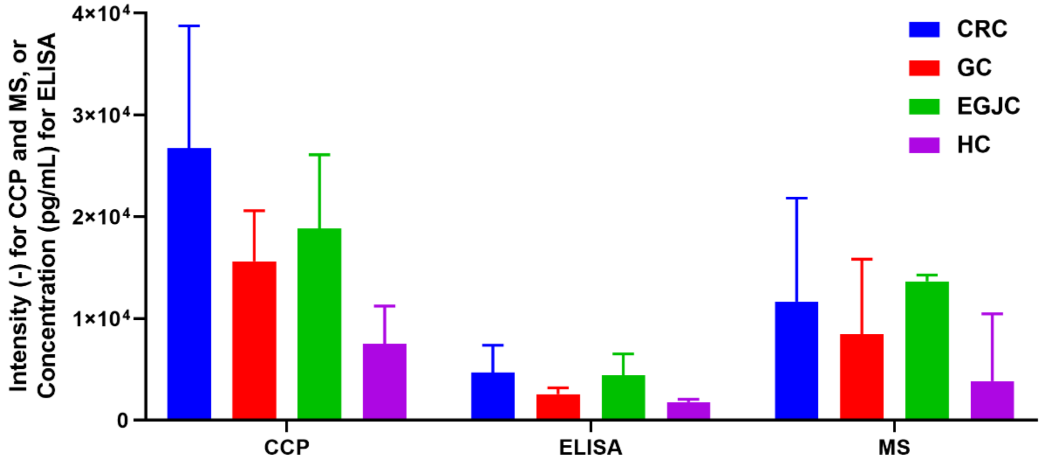

Figure S2: Validation of Plasmin Detection Using a CCP Probe via Enzyme-Linked Immunosorbent Assay and Mass Spectrometry. Plasmin levels in plasma samples from colorectal cancer (CRC), gastric cancer (GC), esophagogastric junction cancer (EGJC), and healthy controls (HC) were assessed using enzyme-linked immunosorbent assay (ELISA; N=5 per group) and mass spectrometry (MS; N=3 per group). These results were compared with plasmin activity profiles obtained using the CCP probe. Both ELISA and MS confirmed elevated plasmin levels in cancer patients, consistent with CCP probe signals and supporting the specificity and biological relevance of CCP-based plasmin detection.
